# Structural Characteristics and Gut Microbiota-Mediated Immunomodulatory Mechanisms of Water- and Alkali-Extracted Polysaccharides from *Tuber indicum*

**DOI:** 10.3390/nu18132202

**Published:** 2026-07-07

**Authors:** Hongfei Ji, Mei Li, Decheng Mao, Yujie Chen, Bing Han, Yanli Zheng, Wenjie Ding, Haiyu Ji

**Affiliations:** 1 School of Life Sciences, Yantai University, Yantai 264005, China; hongfei10052024@163.com (H.J.); lmei9206@163.com (M.L.); decheng0313@163.com (D.M.); xc19554511710@163.com (Y.C.); pharmacology533@163.com (B.H.); 2 Institute of Agricultural Products Preservation and Processing Technology (National Engineering Technology Research Center for Preservation of Agriculture Product), Tianjin Academy of Agricultural Sciences, Tianjin 300384, China; 3 College of Food Science and Engineering, Tianjin University of Science and Technology, Tianjin 300457, China; dingwenjie1120@163.com

**Keywords:** *T. indicum* polysaccharides, structural characteristics, gut microbiota compositions, immunomodulatory activity

## Abstract

Background: *Tuber indicum* is a rare edible and medicinal ectomycorrhizal fungus, and the polysaccharide fractions have attracted extensive research attention owing to their potent immunomodulatory potential. Objective/Methods: To systematically characterize the structural features and gut microbiota-mediated immunoregulatory mechanisms of water-extracted polysaccharide (TIWP) and alkali-extracted polysaccharide (TIAP) from *T. indicum*, two polysaccharide fractions were prepared and comprehensively structurally characterized in this study. Results: The maximum molecular weight of TIWP reached 2.65 × 10^7^ Da, which was dominated by glycosidic linkages of →3)-Glcp-(1→, →2,4)-Glcp-(1→ and →3,6)-Glcp-(1→. TIAP possessed a higher molecular weight up to 3.87 × 10^7^ Da, with predominant glycosidic bonds of →3,6)-Glcp-(1→, →4)-Glcp-(1→ and →2,4)-Glcp-(1→. In vivo bioactivity evaluation results demonstrated that both TIWP and TIAP significantly restored the proportions of CD3^+^ T and CD19^+^ B lymphocyte subsets in peripheral blood and spleen, and alleviated pathological damage in splenic and colonic tissues. Distinct regulatory patterns of gut microbiota were observed between the two polysaccharides: TIWP markedly enriched the genera *Lactobacillus* and *Ruminiclostridium*, whereas TIAP elevated the relative abundances of *Lactobacillus* and *Alloprevotella*. Untargeted metabolomics combined with KEGG pathway enrichment analysis revealed that TIWP and TIAP activated the pantothenic acid and coenzyme A biosynthesis pathway and the linoleic acid metabolism pathway. Conclusions: Collectively, TIWP and TIAP alleviated CTX-triggered immunosuppression through distinct “gut microbiota-metabolite-immunity” regulatory networks due to their structural disparities. This study provides a theoretical basis for the development of gut microecology-targeted functional foods with immunomodulatory functions.

## 1. Introduction

*Tuber indicum* is a rare subterranean mycorrhizal fungus with both edible and medicinal value, attracting extensive research attention due to its unique symbiotic growth characteristics and sophisticated secondary metabolites [1,2]. Prized for its special fragrance, this fungus has long been considered a high-grade edible resource, and its ascocarps are abundant in polysaccharides, proteins, amino acids, ceramides, and aromatic substances [3,4]. *T. indicum* exhibits promising effects in hypoglycemia, anti-allergy, immune regulation, antioxidation, and antitumor activities, among which natural bioactive polysaccharides and peptides have become the research focus [5].

Fungal polysaccharides serve as one of the major bioactive constituents in edible fungi [6]. The biological functions of fungal polysaccharides in vivo exhibit an obvious structure–activity relationship, which is closely related to the monosaccharide composition, polymerization degree, glycosidic linkage mode, and substituent groups [7,8], including antioxidant, antitumor, and immunomodulatory effects [9]. As macromolecular substances, it is hard for fungal polysaccharides to cross the intestinal barrier and enter the blood circulation after oral intake, and their biological activities are mainly realized through regulating gut microbiota metabolism [10].

Dietary polysaccharides can readily regulate the compositions and metabolic functions of gut microbiota, which acts as a critical link connecting dietary ingredients to host health and further affects local and systemic immune status through the gut–immune axis [11]. Under physiological healthy conditions, intestinal commensal bacteria produce a variety of fermentation metabolites, such as short-chain fatty acids, secondary bile acids, and vitamins, which can supply energy to colon cells, sustain the integrity of the intestinal epithelial barrier, and modulate both innate and adaptive immune responses via activating Toll-like receptors and regulating cytokine production [12]. Gut microbiota disturbance disrupts the intestinal barrier, provokes endotoxemia and systemic inflammation, and increases risks of autoimmune diseases and pathogen infections [13]. Accordingly, the gut microbiota–metabolite–immune axis constitutes the core mechanism by which polysaccharides exert their extensive physiological regulatory effects.

Gut microbiota was adopted as the core research entry point, and an integrated research framework combining polysaccharide structural characteristics, gut microecological regulation, and lymphocyte immune response was established in this study to address the research gap regarding structural and immunological disparities of *T. indicum* polysaccharides extracted by different solvents. The water-extracted polysaccharide (TIWP) and alkali-extracted polysaccharide (TIAP) from *T. indicum* were prepared in this study, and the intrinsic structure–activity relationship originating from extraction-triggered structural variations was elucidated. These findings will clarify the differentiated bioactive mechanisms of structurally varied *T. indicum* polysaccharides and supply theoretical and experimental support for developing gut-targeted functional foods and immune intervention products.

## 2. Materials and Methods

### 2.1. Materials and Reagents

*Tuber indicum* was purchased from Hanlu Food Specialty Store (Kunming, China). Anhydrous ethanol was provided by Jinan Ande Biotechnology Co., Ltd. (Jinan, China). T-series dextran standards (T-3, T-10, T-70, T-100, T-500) and monosaccharide standards (rhamnose, arabinose, xylose, mannose, glucose, galactose) were purchased from Solabio Biotechnology Co., Ltd. (Beijing, China). Kunming mice and standard sterile rodent granule feed (main ingredients: corn, wheat, alfalfa grass, fish meal, soybean meal, soybean oil, amino acids, vitamins, and trace elements, such as minerals, which complied with GB14924.1 “General Quality Standard for Laboratory Animal Feed” in China) were provided from Shandong Pengyue Laboratory Animal Technology Co., Ltd. (Jinan, China), and CD19-PE, CD3-FITC monoclonal antibodies were bought from ImmunoWay Biotechnology Company (San Jose, CA, USA). All other chemical reagents were of analytical grade.

### 2.2. Preparation of T. indicum Polysaccharides

**Figure 1:**
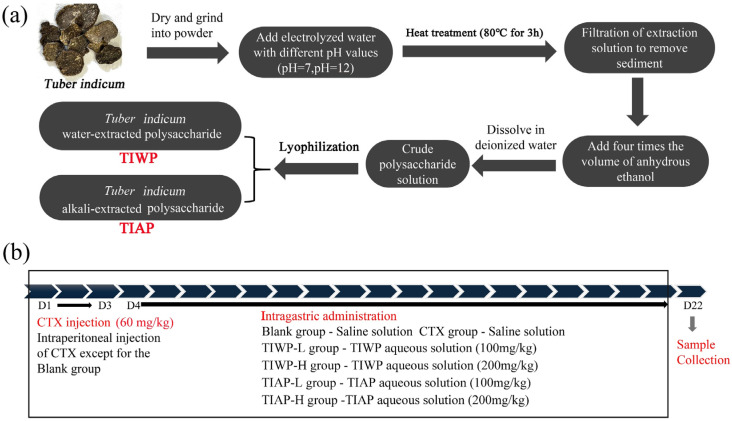
TIWP and TIAP extraction process flow (**a**) and animal experiment design (**b**).

The preparation procedure of *T. indicum* polysaccharides is illustrated in Figure 1a. Dried fruiting bodies of *T. indicum* (100 g) were subjected to water extraction and 1% NaOH alkaline extraction (solid–liquid ratio of 1:20 g/mL in a water bath at 80 °C for 3 h), followed by vacuum concentration and precipitation with 4 volumes of ethyl alcohol. The precipitates were collected and redissolved in deionized water and freeze-dried to obtain polysaccharide fractions, named TIWP (*T. indicum* water-extracted polysaccharide) and TIAP (*T. indicum* alkaline-extracted polysaccharide), respectively. The total carbohydrate/protein contents of TIWP and TIAP were 86.59 ± 4.25%/4.58 ± 0.36% and 89.48 ± 5.72%/4.07 ± 0.29%, respectively, which were determined by phenol-sulfuric acid and the Coomassie brilliant blue method [14].

### 2.3. Molecular Weight Determination of T. indicum Polysaccharides

The molecular weights of TIWP and TIAP were characterized by high-performance gel permeation chromatography (HPGPC) using an Agilent 1200 instrument (Agilent Technologies, Santa Clara, CA, USA) equipped with a TSK-gel G4000PW_xL_ column and a refractive index detector. Sample and dextran standard solutions (1 mg/mL) were filtered through a 0.45 μm membrane and injected, with ultrapure water as the mobile phase at a flow rate of 0.8 mL/min under controlled column temperature (30 °C), detector temperature (35 °C), and injection volume (20 μL). The molecular weights of TIWP and TIAP were finally calculated according to the calibration curve based on dextran standards [15].

### 2.4. Functional Group Determination of T. indicum Polysaccharides

The 0.7 mg of freeze-dried polysaccharide samples were accurately weighed and mixed with dried KBr (150 mg), and then ground thoroughly to a uniform powder and compressed into transparent pellets. Fourier-transform infrared spectroscopy (FT-IR, Bruker, Ettlingen, Germany) was used to record the spectra in the range of 4000–400 cm^−1^ for characterizing the functional groups of TIWP and TIAP [16].

### 2.5. Monosaccharide Composition Determination of T. indicum Polysaccharides

The 10 mg of dried polysaccharide samples were accurately weighed and hydrolyzed with 2 mol/L trifluoroacetic acid at 110 °C for 2 h, and the hydrolysates were concentrated under reduced pressure for acid removal and reconstituted with deionized water to yield monosaccharides. Acetylation was subsequently conducted using acetic anhydride with pyridine as the catalyst, and the acetylated monosaccharide derivatives were isolated via dichloromethane extraction. The derivatives were analyzed by gas chromatography–mass spectrometry (GC-MS, 7000C/US1521U204, Agilent, Santa Clara, CA, USA) equipped with an HP-5 capillary column, and monosaccharide compositions were identified by mass spectral library matching [17].

### 2.6. Glycosidic Bond Configuration Determination of T. indicum Polysaccharides

The glycosidic bond configurations of TIWP and TIAP were determined by modified methylation. The 2.0 mg of polysaccharide samples were dissolved in anhydrous DMSO and NaOH solution, permethylated with methyl iodide, hydrolyzed with 2 mol/L TFA at 110 °C for 2 h, and then the residues were acetylated with acetic anhydride at 100 °C for 1 h, which were dissolved in chloroform, filtered, and then analyzed by GC-MS equipped with an HP-5 capillary column [18].

### 2.7. Animal Experiment Design

As shown in Figure 1b, sixty SPF-grade female Kunming mice (with consistent body weight (18–22 g), no skin lesions, no abnormal activity) were subjected to a one-week acclimatization period. The housing environment was maintained at 22–25 °C and 40–70% humidity, with a 12 h light/dark cycle. Sterile rodent feed and autoclaved tap water were provided ad libitum throughout the entire period, and the food and water were replaced every two days to ensure cleanliness. All experimental procedures were approved by the Ethics Committee of Tianjin University of Science and Technology (approval No. 2025026; date: 26 October 2025). Subsequently, these mice were randomly divided into six groups with 10 mice in each group, namely, the blank group, the cyclophosphamide (CTX) model group, TIWP groups (TIWP-L, 100 mg/kg·d; TIWP-H, 200 mg/kg·d), and TIAP groups (TIAP-L, 100 mg/kg·d; TIAP-H, 200 mg/kg·d). Cage positions were rearranged every 3 days to eliminate location bias; gavage orders were randomized across groups to avoid time-dependent confounding factors. Except for the blank group, all other groups were intraperitoneally injected with CTX at 60 mg/kg for 3 days to establish an immunosuppressive mouse model. Then, the TIWP and TIAP intervention groups were given the corresponding dosages of polysaccharide solution by gavage once daily. The blank and model groups were administered an equal volume of normal saline until day 22. Exclusion criteria (defined before the experiment) were mice with a weight loss of >15% during acclimatization, severe diarrhea, obvious trauma, death before sample collection, and failed tissue/DNA/metabolite extraction. Zero mice were excluded during the whole experiment; all 60 mice survived until sacrifice, with no adverse fatal events recorded. Primary outcomes were body weight, spleen index, and CD3^+^ T/CD19^+^ B lymphocyte proportions. Secondary outcomes were spleen and colon histopathology, gut microbiota composition, differential intestinal metabolites, and KEGG pathway enrichment.

At the end of the experiment, all mice were weighed to record the final body weights. Subsequently, the mice were sacrificed and immediately dissected, and the spleens were isolated and weighed accurately to calculate the spleen indices (the ratios of spleen weights to body weights) [19].

### 2.8. Lymphocyte Subpopulation Distributions Determination

Peripheral blood and spleen tissues were harvested from mice in each group. Erythrocytes were lysed using red blood cell lysis buffer at room temperature, and then the leukocytes were rinsed with normal saline and stained with 2 μL CD19-PE and CD3-FITC antibodies, mixed thoroughly, and incubated in darkness for 20 min to allow antibody-antigen binding. Following saline washing to remove unbound antibodies, leukocytes were resuspended in 500 μL normal saline, and the percentages of CD3^+^ and CD19^+^ lymphocytes were finally analyzed via flow cytometry (Beckman Coulter, Indianapolis, IN, USA) using CytExpert 2.5 software [20].

### 2.9. H&E Staining for Spleen and Colon Tissues

The spleen and colon tissues were collected, rinsed with pre-chilled phosphate-buffered saline, and promptly fixed in 4% paraformaldehyde for 24 h. After fixation, samples were dehydrated with gradient ethanol, cleared in xylene, and embedded in paraffin. Serial 5 μm sections were prepared, and slides were baked at 60 °C for 2 h to strengthen tissue attachment. Paraffin sections were dewaxed and rehydrated before H&E staining, including hematoxylin staining, hydrochloric acid–ethanol differentiation, eosin counterstaining, gradient dehydration, and xylene clearing. All slices were sealed with neutral resin, observed under a light microscope, and photographed for further histological evaluation [15].

### 2.10. 16S rDNA Amplicon Detection

Total genomic DNA was extracted from fecal samples of the blank, model, TIWP-H, and TIAP-H groups. The integrity and purity of extracted DNA were verified via 1% agarose gel electrophoresis. The V3–V4 hypervariable region of the bacterial 16S rRNA gene was amplified using barcode-labeled universal primers, with three independent technical PCR replicates performed for each sample. Amplified PCR fragments were separated on a 2% agarose gel for target fragment purification, and DNA concentration was quantified with the QuantiFluor™-ST blue fluorescence quantification system (Promega Corporation, Fitchburg, WI, USA). Purified amplicons were ligated to Y-shaped sequencing adapters, followed by secondary PCR enrichment. The constructed libraries were denatured with sodium hydroxide to yield single-stranded DNA templates, and qualified libraries were sequenced on the Illumina MiSeq platform (Illumina, Inc., San Diego, CA, USA) adopting the PE250 paired-end sequencing strategy.

Raw sequencing reads were preprocessed by FASTP and FLASH with unified filtering parameters as follows: terminal bases with Phred quality scores below 20 were trimmed using a 50 bp sliding window. Any read with an average quality value lower than 20 within the window was truncated at the window boundary. Reads shorter than 50 bp post-trimming and sequences containing ambiguous N bases were discarded entirely. Paired-end R1 and R2 reads were merged into single consensus sequences according to overlapping regions, with a minimal valid overlap length set to 10 bp. The maximum permissible mismatch rate within overlapping segments was limited to 0.2; sequences failing this criterion were eliminated. Sequences were demultiplexed into corresponding samples based on terminal barcodes and primer sequences, followed by strand orientation correction. Zero mismatches were tolerated for barcode matching, while a maximum of two mismatches was allowed for primer sequences.

After preprocessing, high-quality clean sequences were subjected to two parallel analytical workflows. First, Kraken2 was applied to align all clean reads against the mouse reference genome to separate and quantify host mouse-derived reads and microbial reads, enabling quantitative evaluation of off-target host DNA contamination within the V3-V4 amplicons. Second, non-host microbial sequences were imported into QIIME 2 (version 2023.9) for downstream microbiota profiling. The DADA2 plugin embedded in QIIME 2 was used for sequence denoising, chimera removal, and amplicon sequence variant (ASV) table construction, and taxonomic classification of ASVs was conducted against the SILVA 138 reference database. All raw unprocessed FASTQ sequencing files have been deposited in the NCBI SRA public database. The final filtered valid microbial sequences were adopted for taxonomic annotation and quantitative analysis of intestinal microbiota composition [21].

### 2.11. Gut Microbiota Metabolite Detection

Fecal samples were collected from mice of the model, TIWP-H, and TIAP-H groups and ground into fine powder with liquid nitrogen. Each 100 mg sample was blended with 80% methanol (500 μL), incubated on ice for 5 min, and centrifuged at 15,000× *g* for 20 min at 4 °C. The collected supernatant was used for subsequent LC-MS metabolomics analysis (Dionex Corporation, Chelmsford, MA, USA). Metabolite detection was carried out on a Dionex Ultimate 3000 HPLC system fitted with a 2.1 mm × 100 mm, 1.7 μm C18 column. Trace Finder 3.2.0 software was applied to perform qualitative identification and quantitative determination of differential small-molecule metabolites [22].

### 2.12. Statistical Analysis

All experiments were performed in triplicate; one-way analysis of variance (ANOVA) was conducted with SPSS 26.0. The symbols “*” and “#” were used to mark intergroup differences, with *p* < 0.05 considered statistically significant. All graphs were plotted using raw data exported from professional analytical software or Excel 2021 to intuitively reflect the variation trends and differences among groups.

Each group consisted of 10 samples to ensure sufficient statistical data, while the sample size for microbiota and metabolic indicators was 3, which was the number of mice selected with the proportion of lymphocyte subsets around the average value.

## 3. Results and Discussions

### 3.1. Molecular Weight Distributions of TIWP and TIAP

**Figure 2:**
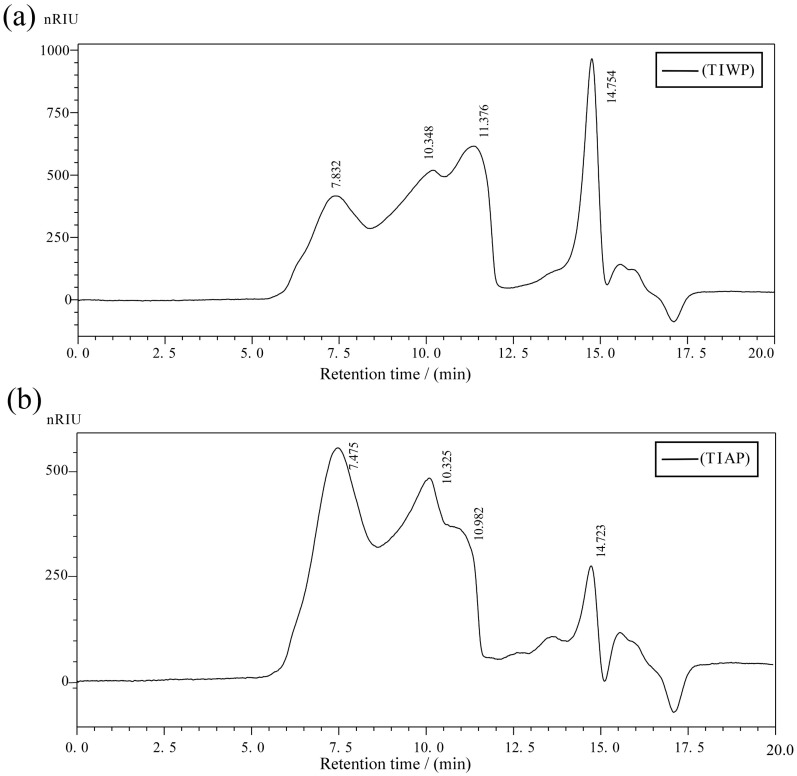
Molecular weight distributions of TIWP (**a**) and TIAP (**b**).

As presented in Figure 2, both samples displayed multiple elution peaks, suggesting that TIWP and TIAP were heterogeneous multicomponent mixtures. Figure 2a shows the elution profile of TIWP. Based on the standard curve equation (y = −0.4628x + 11.0476, R^2^ = 0.9984), where y was the logarithmic molecular weight and x was the retention time, four major peaks at 7.832, 10.348, 11.376, and 14.754 min of TIWP were calculated, and the corresponding molecular weights were about 2.65 × 10^7^, 1.81 × 10^6^, 6.06 × 10^5^, and 1.66 × 10^4^ Da with proportions of 14.41%, 30.03%, 27.03%, and 28.53%, respectively. Figure 2b shows the chromatogram of TIAP, and the molecular weight distributions and proportions are approximately 3.87 × 10^7^ (26.89%), 1.86 × 10^6^ (38.33%), 9.23 × 10^5^ (21.41%), and 1.71 × 10^4^ (13.38%) Da, respectively.

Compared with TIWP, the higher molecular weight distributions of TIAP can be mainly attributed to the differences in extraction medium and structural dissociation mechanism. Under alkaline conditions, the weak hydrogen bonds and intermolecular crosslinking in *Tuber indicum* cell walls can be effectively disrupted, which facilitates the release of high-molecular-weight polysaccharide difficult to dissolve in pure water [23]. Nevertheless, it should be emphasized that the quantitative accuracy will inevitably decline for molecular weight fractions exceeding the upper range of the standard calibration curve.

### 3.2. Characteristic Functional Group Analysis of TIWP and TIAP

**Figure 3:**
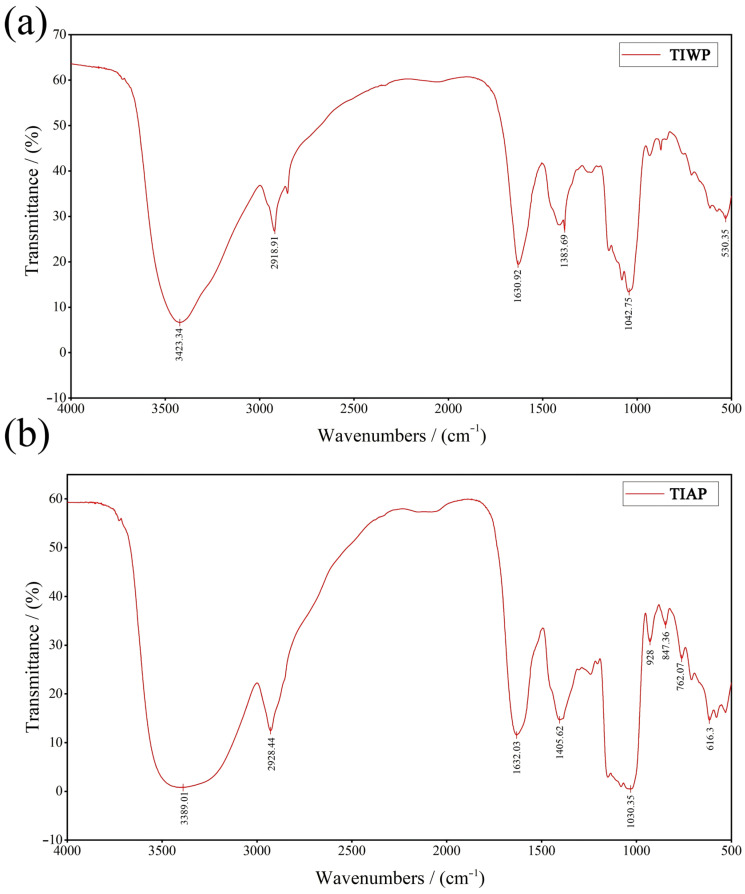
FTIR spectra of TIWP (**a**) and TIAP (**b**).

The signals of TIWP and TIAP over the scanning range of 4000–400 cm^−1^ are presented in Figure 3. As shown, both TIWP and TIAP samples displayed typical polysaccharide characteristic absorption bands: broad O−H stretching at 3423/3389 cm^−1^, C−H stretching of methyl and methylene groups around 2918/2928 cm^−1^, and C−H bending vibration at 1383/1405 cm^−1^ [24,25]. The prominent peaks near 1630 cm^−1^ originated from bound water, while signals at 1000–1250 cm^−1^ were ascribed to the presence of C-O-C and C-O-H linkages [24]. Distinct absorption peaks at 847 cm^−1^ and 928 cm^−1^ confirmed the coexistence of α- and β-glycosidic linkages [26]. Overall, no significant differences were observed in the main functional groups between TIWP and TIAP, while further experiments were still required to elucidate the structural distinctions.

### 3.3. Monosaccharide Compositions of TIWP and TIAP

**Figure 4:**
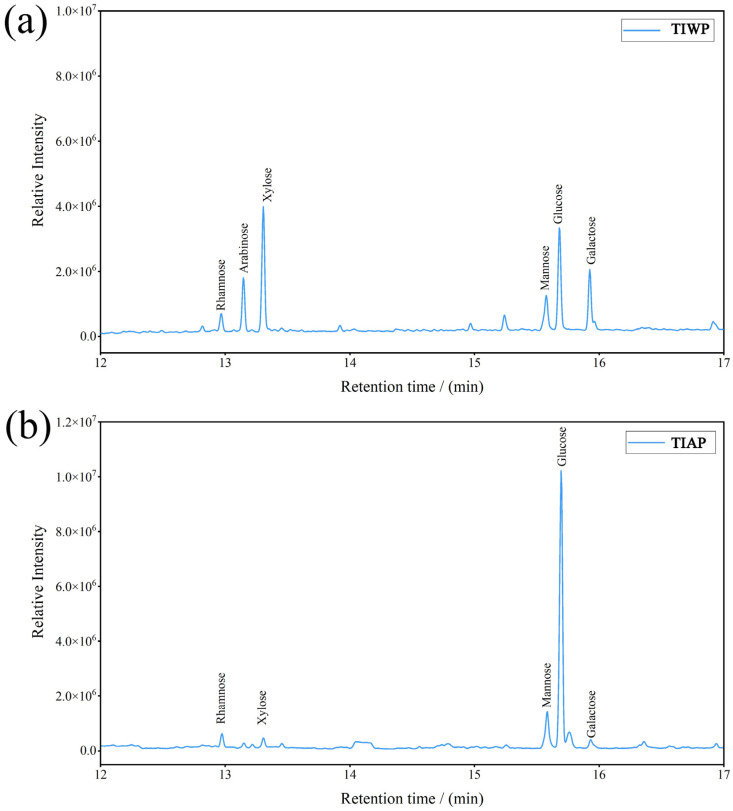
Monosaccharide compositions of TIWP (**a**) and TIAP (**b**).

The monosaccharide compositions differed significantly between TIWP and TIAP, as illustrated in Figure 4. TIWP was composed of Rha, Ara, Xyl, Man, Glc, and Gal, with a molar ratio of 1.00:2.34:5.12:2.15:3.91:2.12, while TIAP mainly contained Man, Glc, and Gal, with a molar ratio of 1.00:4.90:0.41. These compositional discrepancies mainly stemmed from the different extraction solvents: alkaline extraction could cause partial degradation of native polysaccharide backbones and side-chain fragments and facilitate the dissolution and release of more cellulose-derived components, which greatly elevated the relative proportion of Glc [27].

### 3.4. Glycosidic Bond Compositions of TIWP and TIAP

The glycosidic bond distributions of TIWP and TIAP were determined by the methylation method combined with GC-MS, and the results are shown in Table 1. There were 12 types of glycosidic bond configurations detected.

TIWP was dominated by →3)-Glcp-(1→ (13.47%), →2,4)-Glcp-(1→ (11.80%), and →3,6)-Glcp-(1→ (11.38%), with considerable proportions of →4)-Xylp-(1→ (10.44%) and T-Manp-(1→ (10.12%), while TIAP was mainly composed of →3,6)-Glcp-(1→ (16.59%), →4)-Glcp-(1→ (16.16%), and →2,4)-Glcp-(1→ (14.18%), with significantly lower proportions of →4)-Xylp-(1→, →5)-Araf-(1→ and →4)-Galp-(1→ than TIWP, which was consistent with the previous results showing that alkaline extraction could cause partial degradation of polysaccharides and promote the dissolution of more cellulose-derived components.

### 3.5. Physiological Indicator Analysis

**Figure 5:**
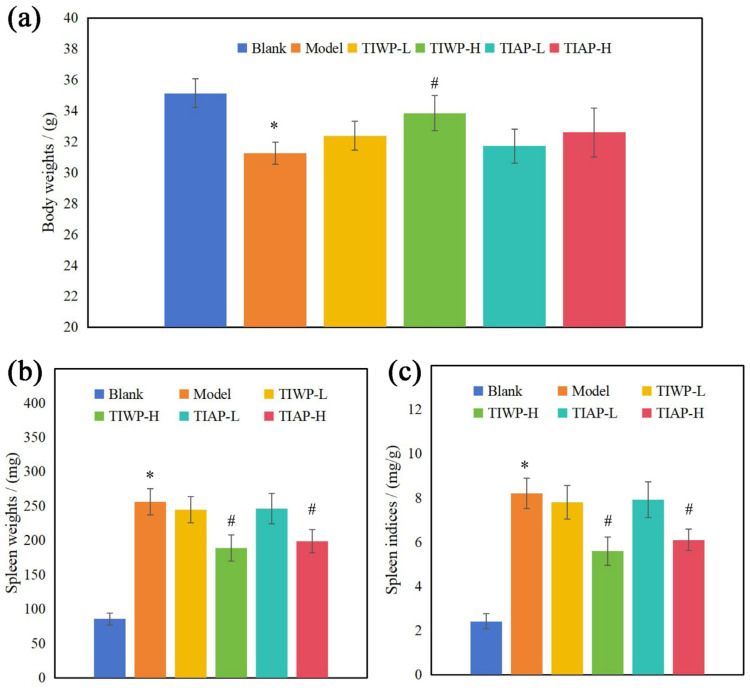
Body weights (**a**) and spleen weights (**b**)/indices (**c**) of mice. Data were presented as mean ± SD, with n = 10 biological replicates per group. Note: *, *p* < 0.05 compared with the blank group; #, *p* < 0.05 compared with the model group.

This study established a CTX-induced immunocompromised mouse model, with TIWP and TIAP administered by intragastric gavage, and body weights as well as spleen weights/indices were determined after the treatment (Figure 5).

Compared with the blank group, the model group showed significant body weight loss and remarkable spleen weights/indices increase, verifying the successful establishment of an immunosuppressive mouse model. Intervention with TIWP and TIAP alleviated body weight reduction and restored spleen weights/indices in a dose-dependent manner, among which the TIWP-H group achieved the optimal recovery and was close to the blank group. These results demonstrated that TIWP and TIAP could effectively ameliorate CTX-triggered immunosuppression via protecting immune organs, and the interaction with gut microbiota deserved in-depth investigation [28].

### 3.6. Peripheral Blood Lymphocyte Subset Analysis

**Figure 6:**
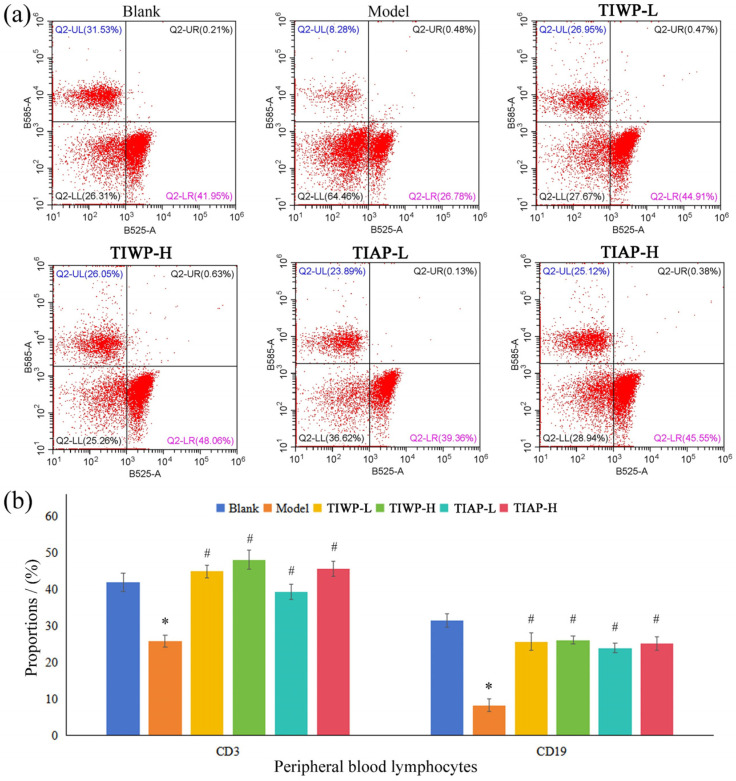
Effects of TIWP and TIAP on the lymphocyte subset distributions in peripheral blood. (**a**) Scatter plot; (**b**) Bar chart. Data were presented as mean ± SD, with n = 10 biological replicates per group. Note: *, *p* < 0.05 compared with the blank group; #, *p* < 0.05 compared with the model group.

To evaluate the effects of TIWP and TIAP on CTX-induced immunosuppression, the proportions of peripheral blood CD3^+^ T and CD19^+^ B lymphocytes were analyzed via flow cytometry (Figure 6). Compared with the blank group, the model group exhibited a significant reduction in both CD3^+^ and CD19^+^ cell proportions (*p* < 0.05), confirming the successful establishment of an immunosuppressive mouse model. After intervention with TIWP and TIAP, the proportions of CD3^+^ and CD19^+^ lymphocytes were significantly restored in all dose groups (*p* < 0.05). Notably, the TIWP-H group showed the most pronounced recovery effects, with CD3^+^ T cell proportions even slightly exceeding the baseline level of the blank group, followed by the TIAP-H group. These data indicated that both TIWP and TIAP could effectively reverse CTX-induced depletion of T and B lymphocytes in a dose-dependent manner.

The balance of T and B lymphocytes is critical for maintaining normal immune function, and CTX is known to disrupt this balance by inducing lymphocyte apoptosis and inhibiting their proliferation [29]. In the present study, CTX treatment significantly reduced the proportions of both CD3^+^ T cells and CD19^+^ B cells, which was consistent with previous reports of chemotherapy-induced immunosuppression. The intervention with TIWP and TIAP significantly restored the levels of these lymphocyte subsets, suggesting that the two polysaccharides could ameliorate the impaired cellular and humoral immunity caused by CTX. The superior effects of TIWP-H over TIAP-H might be attributed to differences in their structural characteristics, such as molecular weight, monosaccharide composition, or branching degree, which were known to influence the immunomodulatory activity of polysaccharides [30].

### 3.7. Spleen Lymphocyte Subset Analysis

**Figure 7:**
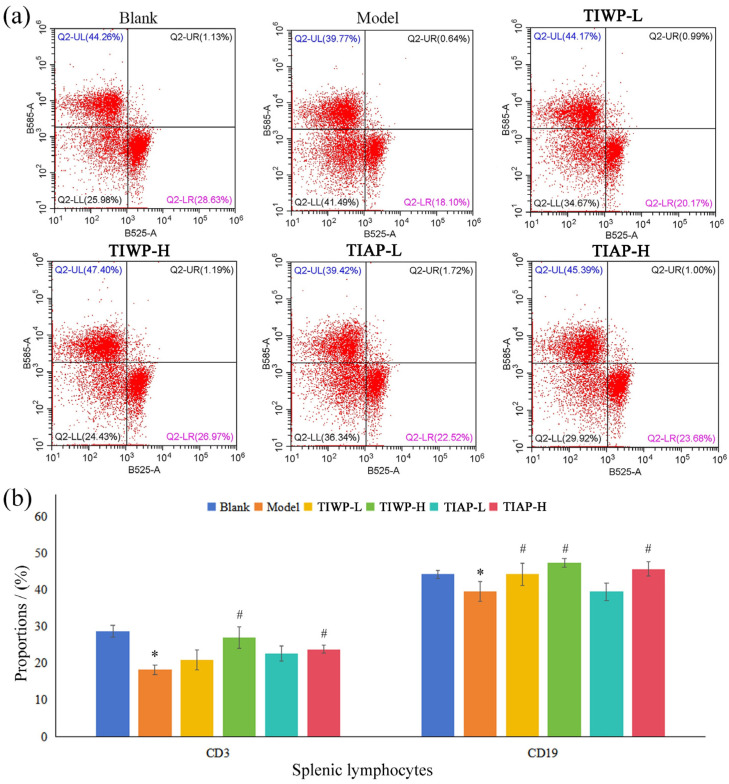
Effects of TIWP and TIAP on the lymphocyte subset distributions in spleens. (**a**) Scatter plot; (**b**) Bar chart. Data were presented as mean ± SD, with n = 10 biological replicates per group. Note: *, *p* < 0.05 compared with the blank group; #, *p* < 0.05 compared with the model group.

To further investigate the immunomodulatory effects of TIWP and TIAP on the primary immune organ, the proportions of splenic CD3^+^ T lymphocytes and CD19^+^ B lymphocytes using flow cytometry were analyzed (Figure 7). As shown, compared with the blank group, the model group exhibited a significant reduction in both CD3^+^ T cells and CD19^+^ B cells (*p* < 0.05), confirming that CTX induced severe impairment of splenic immune cell populations. Following intervention with TIWP and TIAP, the proportions of CD3^+^ and CD19^+^ lymphocytes were significantly restored (*p* < 0.05). Specifically, the TIWP-H group showed the most pronounced recovery effects. These data indicated that both TIWP and TIAP could effectively reverse CTX-induced depletion of splenic T and B lymphocytes, with TIWP showing superior efficacy, particularly at high dosage.

The spleen, as the largest secondary immune organ, is critical for maintaining adaptive immunity through mature T and B lymphocytes [31]. The significant reduction in splenic CD3^+^ T and CD19^+^ B cells in the CTX-treated model group observed in this study was consistent with the peripheral blood findings, further confirming the establishment of a systemic immunosuppressive state. Intervention with TIWP and TIAP effectively restored the proportions of both T and B lymphocytes in the spleen, mirroring the recovery trend in peripheral blood and indicating that their immunomodulatory effects extend beyond circulating immune cells to improve overall immune organ function.

### 3.8. Organization Structure Analysis of the Spleen and Colon

**Figure 8:**
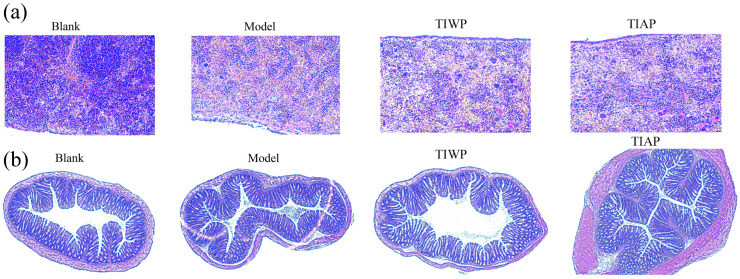
H&E staining results of mouse spleen (**a**) and colon (**b**). Data were presented as mean ± SD, with n = 10 biological replicates per group (scale bar = 20 µm).

Representative H&E-stained sections of spleen tissues were examined to assess histological architecture and cell distributions across groups, and the results are displayed in Figure 8a. The blank group exhibited intact splenic tissue with dense, well-organized lymphocytes with a clear boundary between the red pulp and the white pulp, indicating a normal immune microenvironment. In contrast, the CTX-induced model group displayed significant lymphocyte depletion with unclear organizational boundaries, indicating severe immune tissue damage. Both TIWP and TIAP polysaccharide interventions partially restored splenic structure, with improved red/white pulp boundary compared with the model group, demonstrating superior protective effects on splenic immune tissue.

Figure 8b exhibited the H&E staining results of mouse colon tissues to evaluate the protective effects of polysaccharides on intestinal mucosal integrity and histological morphology. The blank group presented well-preserved colonic mucosa with neatly arranged crypt structures, abundant goblet cells, and minimal inflammatory infiltration. The model group showed marked pathological damage, including mucosal thinning, shortened and disorganized crypts, reduced goblet cell counts, and extensive inflammatory cell infiltration in the lamina propria. TIWP and TIAP interventions alleviated these pathological changes, with restored mucosal thickness, improved crypt arrangement, and decreased inflammatory cell infiltration compared with the model group, indicating effective protection against CTX-induced intestinal injury.

The present histopathological findings from spleen and colon tissues further confirmed the toxic effects of CTX, demonstrating the ability to disrupt both systemic immune function and intestinal mucosal integrity [32]. Notably, TIWP and TIAP partially reversed the pathological alterations, exhibiting superior efficacy in restoring splenic cellularity, reconstructing lymphoid tissue architecture, and preserving colonic epithelial integrity, which was consistent with the relevant research [33].

### 3.9. Effects of TIWP and TIAP on Gut Microbiota Compositions

**Figure 9:**
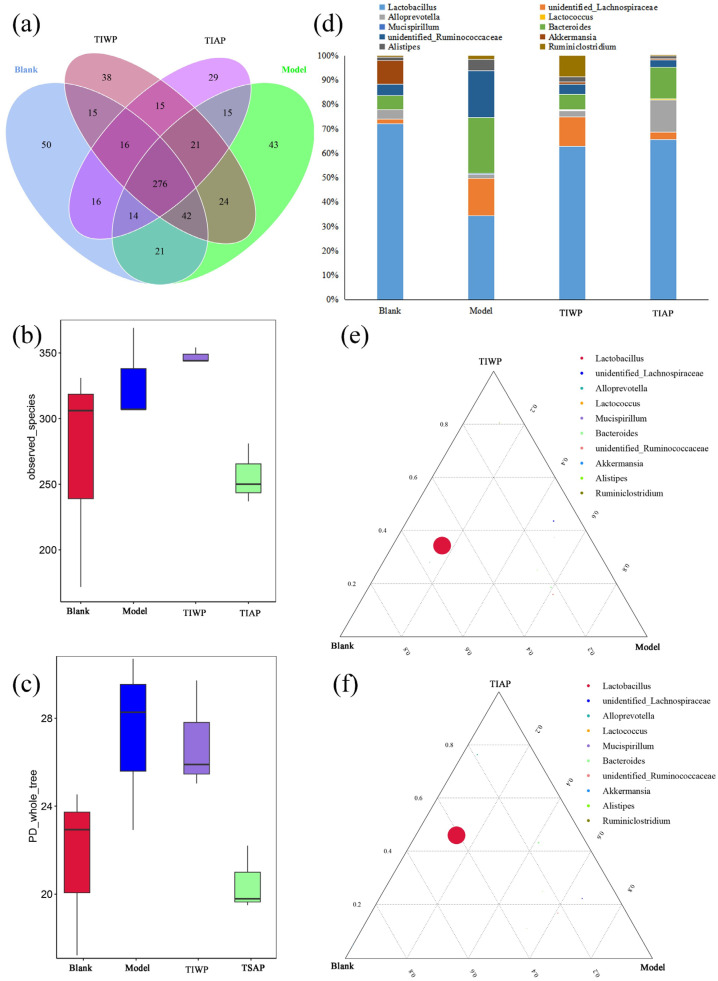
Effects of TIWP and TIAP on gut microbiota diversity and composition in mice, including OTU distributions (**a**), α-diversity indices (**b**,**c**), top 10 genus-level relative abundances (**d**), and ternary phase diagrams of dominant genera (**e**,**f**). Data were presented as mean ± SD, with n = 3 biological replicates per group.

The histological results revealed that CTX-induced colonic damage was significantly ameliorated following polysaccharide intervention, implying potential concurrent changes in the gut microbiota—a key regulator of intestinal homeostasis [34]. Therefore, 16S rDNA high-throughput sequencing targeting the V3–V4 region was performed on fecal samples from all groups to analyze gut microbiota diversity and composition, with the results presented in Figure 9, and the SRA records are accessible at the following link: https://www.ncbi.nlm.nih.gov/sra/PRJNA1481606 (Temporary Submission ID SUB16268850, accessed on 24 June 2026).

First, the core microbiota shared across groups was visualized via a Venn diagram (Figure 9a). A total of 276 OTUs were common to all four groups, while the blank, model, TIWP, and TIAP groups harbored 50, 43, 38, and 29 unique OTUs, respectively, indicating distinct microbial community characteristics under different conditions. α-diversity analysis further revealed that the observed species index (Figure 9b) and PD whole tree index (Figure 9c) were significantly altered in the CTX-induced model group, with a marked increase in community richness and phylogenetic diversity relative to the blank group. Notably, TIWP treatment restored these indices to levels close to the blank control, while the TIAP group showed lower diversity than the model group, suggesting differential regulatory effects of the two polysaccharides on microbial community complexity.

At the genus level, stacked bar plots (Figure 9d) illustrated the relative abundance of dominant bacteria across groups. The blank and both polysaccharide-treated groups exhibited high relative abundance of *Lactobacillus*, which was notably reduced in the model group. Compared with the model group, the TIWP group showed significant enrichment of beneficial genera, including *Lactobacillus* and *Ruminiclostridium*, while the TIAP group displayed increased abundance of *Lactobacillus* and *Alloprevotella*. Ternary phase diagrams (Figure 9e,f) further confirmed these patterns, showing that *Lactobacillus* was preferentially enriched in the blank and polysaccharide-treated groups. Collectively, these findings indicate that both TIWP and TIAP interventions effectively reversed CTX-induced gut microbiota dysbiosis, particularly by restoring the abundance of beneficial bacteria within the *Lactobacillus* genus, indicating enhanced immune functions in hosts [35]. Notably, TIWP preferentially enriched *Ruminiclostridium* associated with energy metabolism and short-chain fatty acid production, whereas TIAP modulated mucosal immunity and inflammatory responses by increasing *Alloprevotella* abundances [36]. This distinct microbial regulation pattern provided key mechanistic evidence explaining how the two polysaccharides synergistically improve host immune functions through different gut microbiota-dependent pathways. Further comprehensive integration with metabolomics data is required to fully elucidate the underlying regulatory mechanisms.

### 3.10. Effects of TIWP and TIAP on Gut Microbiota Metabolism

The small-molecule metabolites present in the intestines of mice from each group following TIWP and TIAP gavage interventions were analyzed and identified using liquid chromatography–mass spectrometry (LC-MS). The results are presented in Supplementary Tables S1 and S2, respectively.

As shown in Supplementary Table S1, compared with the model group, a total of 468 metabolites exhibited significant differential expression (*p* < 0.05) in the intestines of TIWP-treated mice, among which 134 were downregulated and 334 were upregulated. Among all upregulated metabolites, peptides and amino acid derivatives represented the most abundant category in both variety and quantity. This overall upregulation suggests the activation of protein metabolic networks. Within this dominant category, in addition to various dipeptides (e.g., Tryptophyl-Valine) and tripeptides (e.g., Ala-Ile-Leu), the increase in the tryptophan-derived microbial metabolite—indolelactic acid—is of particular interest [37]. This molecule is a key active metabolite derived from the microbial metabolism of tryptophan in the gut. By activating the aryl hydrocarbon receptor (AhR) pathway, it directly enhances intestinal epithelial barrier function and promotes regulatory T cell (Treg) differentiation, thereby serving as a critical signaling molecule within the gut–microbiota–immune axis [38]. The upregulation of this metabolite suggests that TIWP may enhance this protective metabolic pathway by modulating microbiota function. Concurrently, the accumulation of numerous small peptides may reflect several underlying physiological changes, including improved efficiency of intestinal protein digestion and absorption, increased protein turnover during tissue repair, and/or enhanced proteolytic activity of the gut microbiota.

Furthermore, a diverse array of lipids and their metabolites was observed. From the initiation of inflammation to the synthesis of protective mediators, the level of arachidonic acid (AA)—a central precursor—was significantly increased, thereby providing abundant substrates for the subsequent production of lipid mediators [39]. However, a more critical finding was the concurrent increase in 11,12-epoxyeicosatrienoic acid (11(12)-EET). In vivo, arachidonic acid (AA) is primarily metabolized via two competing pathways: the cyclooxygenase/lipoxygenase pathway generates pro-inflammatory mediators, such as prostaglandins and leukotrienes; the cytochrome P450 pathway produces anti-inflammatory and tissue-protective EETs [40,41]. The upregulation of 11(12)-EET suggests that TIWP intervention may shift AA metabolism towards the P450/EET branch, which is cytoprotective and anti-inflammatory [42]. This metabolic shift is consistent with the observed phenotypic improvements, including the restoration of colonic mucosal structure and the recovery of splenic lymphocyte numbers.

Of particular significance is the direct detection of elevated levels of pantothenic acid (vitamin B5) among the metabolites. Pantothenic acid is the sole precursor for the synthesis of coenzyme A (CoA), which acts as a central cofactor in key biochemical processes [43]. These include, but are not limited to, cellular energy metabolism (the tricarboxylic acid cycle), fatty acid activation and oxidation, and amino acid metabolism [44]. This finding provides empirical evidence at the metabolite level for the activation of the “Pantothenic acid and CoA biosynthesis” pathway identified in KEGG enrichment analysis.

In summary, TIWP has been demonstrated to enhance intestinal barrier function and reduce inflammation by promoting the production of protective metabolites, regulating lipid metabolic pathways, and enhancing beneficial microbial metabolism, thereby restoring immune homeostasis.

Supplementary Table S2 presents a comprehensive list of the 99 metabolites that were found to be significantly expressed in the TIAP group in comparison to the model group, with 35 metabolites of upregulation and 64 metabolites of downregulation. In a manner analogous to the list of metabolites that were found to be upregulated after TIWP intervention, metabolites that were found to be upregulated after TIAP intervention can also be categorized into three major groups: peptides/amino acid derivatives, lipids and their metabolites, and drugs/exogenous compounds.

The functional significance of elevated core metabolites is evident: indole, a classic aryl hydrocarbon receptor (AhR) ligand produced by gut microbiota metabolism of dietary tryptophan, exhibited significantly increased levels. The aryl hydrocarbon receptor (AhR) is a pivotal transcription factor that links the microbiota to host immunity. Its activation has been shown to directly fortify the intestinal barrier, promote immune tolerance, and stimulate the secretion of protective factors [45]. Linoleic acid and its metabolites, 9-Oxo-ODE and 13-HODE, are also critically important upregulated metabolites. Linoleic acid is an essential polyunsaturated fatty acid that serves as a key component of phospholipids in cell membranes and as a precursor for the biosynthesis of longer-chain omega-6 fatty acids, such as arachidonic acid [46]. Linoleic acid derivatives, 9-HODE and 13-HODE, are natural ligands for PPARγ. Activation of PPARγ effectively suppresses the NF-κB signaling pathway, reducing the production of pro-inflammatory cytokines, such as TNF-α and IL-6, as demonstrated in intestinal inflammation models [47,48]. The widespread upregulation of peptides was a common feature observed in both polysaccharide interventions, suggesting that *T. indicum* polysaccharides—whether extracted with water or alkali—strongly promote protein/amino acid metabolism processes within the gut.

In summary, TIWP demonstrates superior metabolic remodeling capabilities compared to TIAP. It not only supplies arachidonic acid (AA) but also efficiently directs its metabolism to produce protective 11(12)-EET, thereby activating a more comprehensive anti-inflammatory and repair program. In contrast, TIAP exhibits lower efficiency in guiding such core lipid metabolic networks, which may contribute to its weaker systemic immunomodulatory effects.

**Figure 10:**
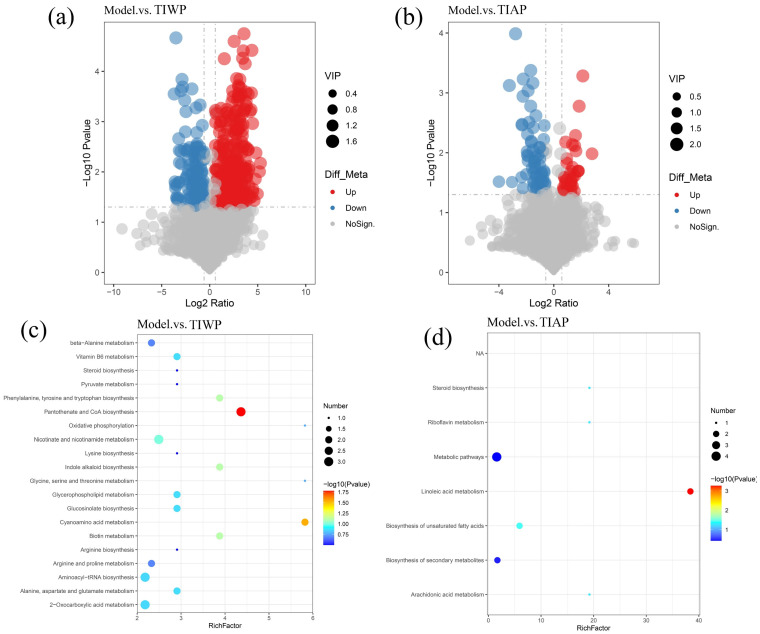
Volcano plots of differential metabolites ((**a**) TIWP; (**b**) TIAP) and their corresponding KEGG pathway enrichment bubble diagrams ((**c**) TIWP; (**d**) TIAP). The bubble color gradient from blue to red represents the magnitude of −log_10_(*p*-value), with red corresponding to smaller *p*-values. Data were presented as mean ± SD, with n = 3 biological replicates per group.

In the volcano plots in Figure 10a,b, each dot represents one metabolite. Compared with the model group, significantly upregulated and downregulated metabolites were labeled in red and blue, respectively, while metabolites with no significant difference were shown in gray. These distribution patterns were consistent with the metabolite variation trends presented in Supplementary Tables S1 and S2.

Subsequently, KEGG pathway enrichment analysis was performed on the screened differential metabolites (*p* < 0.05, FDR < 0.05), and the results are displayed in Figure 10c,d. The color gradient of bubbles from blue to red indicated the enrichment significance of each metabolic pathway. In the TIWP group, the pantothenic acid and coenzyme A biosynthesis pathways (mediated by C358-Pantothenic acid, C1756-Panthenol, C3075-L-Aspartic acid) exhibited the most remarkable enrichment. The activation of this pathway was in line with the elevated pantothenic acid level identified in metabolite profiling, as well as the metabolic tendency that facilitates the conversion of arachidonic acid to the protective metabolite 11(12)-EET. Collectively, these results suggested that TIWP might accelerate cellular repair and trigger immune activation by modulating this core energy metabolism and biosynthesis hub [43].

In contrast, the TIAP group exhibited specific activation of the linoleic acid metabolism pathway (mediated by C599-Linoleic acid and C1464-Arachidonic acid). This result agreed with the observation that TIAP hardly elevated the production of 11(12)-EET, yet it markedly enriched *Alloprevotella*. It was thus inferred that the immunomodulatory effects of TIAP were more dependent on the regulation of gut microbiota. By modulating linoleic acid metabolism, TIAP facilitated the generation of immunomodulatory lipids including hydroxyoctadecadienoic acid, consequently exerting potent anti-inflammatory and immunoregulatory activities [49].

In summary, KEGG analysis confirmed at the pathway level that the two polysaccharides exhibited distinct mechanisms of action: TIWP tended to activate central anabolic pathways (pantothenic acid/CoA pathway), while TIAP focused on regulating specific immunomodulatory lipid metabolism axes (linoleic acid pathway). This provided pathway-level evidence that both compounds improved immunosuppressed states through distinct “microbiome-metabolism-immunity” networks.

## 4. Conclusions

In conclusion, the effective immunomodulatory activities against CTX-induced immunosuppression in mice were verified for TIWP and TIAP with divergent structural features in this study. The maximum molecular weight of TIWP was determined to be 2.65 × 10^7^ Da, and the glycosidic linkages were predominantly composed of →3)-Glcp-(1→ (13.47%), →2,4)-Glcp-(1→ (11.80%) and →3,6)-Glcp-(1→ (11.38%). A higher maximum molecular weight of 3.87 × 10^7^ Da was detected for TIAP, which was dominated by →3,6)-Glcp-(1→ (16.59%), →4)-Glcp-(1→ (16.16%) and →2,4)-Glcp-(1→ (14.18%). Additionally, TIWP and TIAP exhibited significant recoveries of CD3^+^ T and CD19^+^ B lymphocyte subsets in peripheral blood and spleen and triggered distinct gut microbiota remodeling patterns, such as TIWP enriching *Lactobacillus* and *Ruminiclostridium*, while TIAP enriched *Lactobacillus* and *Alloprevotella*. Furthermore, separated metabolic cascades were activated by these two fractions; the pantothenate and coenzyme A biosynthesis pathway was modulated by TIWP, while linoleic acid metabolism was regulated by TIAP. These findings clearly clarify the structure–activity relationship and specific immunomodulatory mechanisms of *T. indicum* polysaccharides, further enriching the studies on edible fungal polysaccharides and providing a solid theoretical and experimental basis for the development of gut microbiota-targeted functional foods and clinical immune intervention strategies.

However, several limitations of this study are still acknowledged. First, only polysaccharide samples extracted in the laboratory are analyzed, and no commercially available polysaccharide preparations (e.g., *Astragalus* polysaccharides) are incorporated as positive control groups for horizontal efficacy comparison, so the relative immunomodulatory potency of TIWP and TIAP against mainstream bioactive polysaccharides cannot be quantitatively evaluated. Second, all in vivo assays are performed on CTX-induced immunosuppressive mice, while the immunoregulatory effects of TIWP and TIAP are not validated under other immune disorder models or in vitro conditions, which restricts the generalizability of the observed biological effects. Third, the interactions between polysaccharides and key differential metabolites or core intestinal beneficial bacteria are only speculated based on multi-omics correlation analysis, and direct cell-level or in vitro co-culture verification experiments are not conducted to reveal the precise molecular binding targets of TIWP and TIAP.

## Figures and Tables

**Table 1:** Glycosidic bond distributions and proportions in TIWP and TIAP.

No.	Retention Time/(min)	Glycosidic Bond Configuration	Derivative Abbreviations	Main Ion Fragment (*m*/*z*)	TIWP Proportions (%)	TIAP Proportions (%)
1	7.06	→2)-Rha*p*-(1→	3,4-Me_2_-Rha*p*	43, 57, 85, 99, 141, 155	6.89	5.00
2	7.97	T-Glc*p*-(1→	2,3,4,6-Me_4_-Glc*p*	43, 71, 85, 99, 127, 155	4.13	4.34
3	10.51	→4)-Xyl*p*-(1→	2,3-Me_2_-Xyl*p*	43, 57, 85, 99, 127, 155	10.44	6.31
4	10.92	→3)-Glc*p*-(1→	2,4,6-Me_3_-Glc*p*	41, 57, 107, 135, 163, 191, 206	13.47	14.27
5	11.50	→5)-Ara*f*-(1→	2,3-Me_2_-Ara*f*	43, 57, 71, 85, 99, 127, 155	7.74	2.49
6	12.15	→4)-Glc*p*-(1→	2,3,4-Me_3_-Glc*p*	43, 57, 79, 119, 161, 176	2.55	16.16
7	15.39	→4)-Gal*p*-(1→	2,3,6-Me_3_-Gal*p*	43, 57, 85, 109, 123, 165, 221, 277	9.65	1.63
8	15.79	→3)-Gal*p*-(1→	2,4,6-Me_3_-Gal*p*	43, 71, 85, 99, 127, 155	3.73	4.17
9	20.14	T-Man*p*-(1→	2,3,4,6-Me_4_-Man*p*	43, 71, 87, 117, 129, 161, 173, 207	10.12	6.54
10	24.52	→2,6)-Man*p*-(1→	3,4-Me_2_-Man*p*	43, 57, 85, 151, 247, 305, 361	8.11	8.32
11	24.74	→3,6)-Glc*p*-(1→	2,4-Me_2_-Glc*p*	41, 57, 76, 104, 149, 178, 205, 223	11.38	16.59
12	25.32	→2,4)-Glc*p*-(1→	3,6-Me_2_-Glc*p*	43, 57, 73, 115, 129, 157, 185, 213, 256	11.80	14.18

## Data Availability

The raw data supporting the conclusions of this article will be made available by the authors on request. SRA records of the 16s rDNA results are accessible at the following link: https://www.ncbi.nlm.nih.gov/sra/PRJNA1481606, accessed on 24 June 2026.

## Supplementary Materials

The following supporting information can be downloaded at: https://www.mdpi.com/article/10.3390/nu18132202/s1, Supplementary Table S1 Differential metabolites information of TIWP group compared with model group; Supplementary Table S2 Differential metabolites information of TIAP group compared with model group.
