# Supplementary material for: Structural Characteristics and Gut Microbiota-Mediated Immunomodulatory Mechanisms of Water- and Alkali-Extracted Polysaccharides from Tuber indicum
Source: Nutrients. 2026 Jul 7;18(13):2202. doi: 10.3390/nu18132202 (PMC13363891; doi:10.3390/nu18132202)
Supplement: Supplementary file 1 [file nutrients-18-02202-s001.zip › nutrients-4380171-supplementary.pdf]

Supplementary Table S1. Differential metabolites information of TIWP group compared with model group.

ID	Description	Retention_time (min)	m/z	FC	log2FC	Pvalue	Up.Down
C17	3-methyl-3,4-dihydro-1H-1,4-benzodiazepine-2,5-dione	0.96	190.08	2.75	1.46	0.01813	up
C25	LPC 18:1-SN1	7.39	521.35	1.74	0.80	0.01351	up
C26	3,4-Tetramethylene-pyrrolidinobutiophenone	3.36	271.19	5.74	2.52	0.00581	up
C35	2-{2-[2-(Octanoyloxy)ethoxy]ethoxy}ethyl decanoate	7.53	430.33	4.98	2.32	0.01237	up
C40	Creatine	0.81	131.07	2.93	1.55	0.01373	up
C55	NP-016455	2.86	242.13	6.66	2.73	0.02086	up
C56	4alpha-Carboxy-5alpha-cholesta-8-en-3beta-ol	11.10	430.34	3.01	1.59	0.04722	up
C57	Mirtazapine-d3	4.50	268.18	4.36	2.12	0.04542	up
C66	Tryptophyl-Valine	3.44	303.16	3.27	1.71	0.00067	up
C73	Isouron	0.75	211.13	3.33	1.73	0.01362	up
C76	2-Pyrrolidinone	0.65	85.05	2.01	1.01	0.03717	up
C99	Creatinine	0.56	113.06	2.60	1.38	0.02652	up
C102	Lysylproline	2.91	243.16	7.92	2.98	0.00028	up
C116	2-(Cyclohexylmethylidene)-1,2,3,4-tetrahydronaphthalen-1-one	3.74	240.15	4.97	2.31	0.01193	up
C117	(+/-)-Cannabichromeorcin	3.74	258.16	4.97	2.31	0.01193	up
C118	Phenyltoloxamine	2.07	255.16	3.91	1.97	0.02556	up
C119	Glu-Leu	2.90	260.14	5.78	2.53	0.01339	up
C124	PC O-18:1	7.26	521.35	1.77	0.82	0.01268	up
C130	PPK	3.20	340.21	6.18	2.63	0.00975	up
C135	1,4-Anhydro-2-(cyclobutylamino)-2,5-dideoxy-5-[(isopropylcarbamoyl)amino]-D-arabinitol	3.01	271.19	4.39	2.13	0.01628	up
C143	Difenzoquat	0.96	248.13	2.47	1.31	0.04203	up
C152	Amobarbital	3.49	226.13	2.72	1.44	0.01707	up
C156	N-Arachidonoyl taurine	3.25	411.25	12.09	3.60	0.02717	up
C163	1,4-Anhydro-2,5-dideoxy-5-[(isopropylcarbamoyl)amino]-2-(4-piperidinylamino)-D-arabinitol	1.35	300.22	8.26	3.05	0.02999	up
C193	1-Cyclohexyl-3-[(1S,2S,3S,4R,5R)-3-hydroxy-4-(1-piperidinyl)-6,8-dioxabicyclo[3.2.1]oct-2-yl]urea	4.87	353.23	8.04	3.01	0.04110	up
C205	Ile-Leu	3.43	244.18	5.03	2.33	0.01827	up
C210	NP-020439	3.54	242.13	4.16	2.06	0.00752	up
C234	LPC 20:4-SN1	6.89	543.33	3.45	1.78	0.00057	up
C245	Pro-Pro	3.09	212.12	3.51	1.81	0.00066	up

C248	3-[(4-hydroxyphenyl)methyl]-octahydropyrrolo[1,2-a]pyrazine-1,4-dione	3.66	260.12	2.84	1.51	0.00941	up
C274	SLK	3.45	346.22	3.16	1.66	0.02995	up
C277	(S)-[(1S,2R,4S,5R)-5-ethyl-1-azabicyclo[2.2.2]octan-2-yl](6-methoxyquinolin-4-yl)methanol	1.48	326.20	5.59	2.48	0.00292	up
C281	Acetylcholine	2.82	145.11	2.02	1.02	0.00160	up
C289	LPK	3.83	356.24	5.78	2.53	0.00075	up
C293	Ajmaline	3.37	326.20	3.25	1.70	0.00626	up
C300	Lithocholic acid glycine conjugate	6.24	433.32	3.40	1.77	0.00436	up
C308	Glu-Val-Leu	3.58	359.21	8.82	3.14	0.00099	up
C314	N1-[4-(2-nitrovinyl)phenyl]acetamide	1.10	206.07	3.99	2.00	0.00079	up
C316	N-Acetyl-DL-tryptophan	3.06	246.10	6.80	2.76	0.00631	up
C331	CAY10401	3.96	382.26	16.07	4.01	0.00028	up
C333	Norbuprenorphine	3.80	413.26	10.71	3.42	0.00005	up
C334	GPK	2.83	300.18	7.54	2.91	0.00908	up
C335	Tri-o-Cresyl phosphate	7.87	368.12	2.79	1.48	0.00246	up
C341	Gly-Met	0.55	206.07	4.44	2.15	0.00094	up
C342	1,4-Anhydro-5-[(cyclopropylcarbonyl)amino]-2,5-dideoxy-2-(3-oxetanylamino)-D-arabinitol	3.41	256.14	4.05	2.02	0.01506	up
C347	Val-Leu	3.04	230.16	11.32	3.50	0.04124	up
C351	Ranolazine	3.55	427.24	6.66	2.74	0.00124	up
C353	NP-023108	2.97	243.13	6.48	2.70	0.01544	up
C356	N-(3-acetamidopropyl)pyrrolidin-2-one	0.84	184.12	1.86	0.90	0.04326	up
C357	SPK	1.36	330.19	21.44	4.42	0.03153	up
C358	Pantothenic acid	0.82	219.11	1.81	0.86	0.04540	up
C363	Val-Gln-Arg	5.25	401.23	5.96	2.57	0.01100	up
C367	{(3R,4S)-1-(Cyclopropylcarbonyl)-3-[2-(4-phenyl-1-piperazinyl)ethyl]-4-piperidinyl}acetic acid	3.44	399.25	8.16	3.03	0.00021	up
C388	MN-25	3.99	439.28	17.10	4.10	0.02984	up
C390	Valylvaline	3.04	216.15	4.28	2.10	0.02705	up
C397	PC O-18:2	6.77	519.33	2.65	1.40	0.00347	up
C400	RNK	4.15	416.24	7.84	2.97	0.00032	up
C413	Leu-Leu	3.84	244.18	4.64	2.22	0.00154	up
C423	{(3R,4S)-1-(Cyclobutylcarbonyl)-3-[2-(4-phenyl-1-piperazinyl)ethyl]-4-piperidinyl}acetic acid	3.03	413.26	12.65	3.66	0.01815	up
C424	NP-016141	3.71	355.21	4.83	2.27	0.00587	up
C428	LPC 20:3-SN1	7.16	545.35	2.39	1.26	0.00451	up
C430	8-Amino-7-oxononanoic acid	1.69	187.12	2.96	1.57	0.01045	up

C431	Cyclo(Ile-Ala)	3.48	184.12	2.68	1.42	0.00753	up
C441	3 Amino-phenylpropionic acid	1.14	165.08	2.83	1.50	0.04221	up
C450	6-[(6-Aminohexanoyl)amino]hexanoic acid	3.22	244.18	3.72	1.89	0.03951	up
C456	Indoline	3.21	119.07	3.96	1.98	0.01772	up
C470	1-[(1S,2S,3S,4R,5R)-4-(4-Benzyl-1-piperidinyl)-3-hydroxy-6,8-dioxabicyclo[3.2.1]oct-2-yl]-3- [illegible]	2.75	443.27	36.45	5.19	0.00989	up
C483	Ile-Ser-Pro	3.35	315.18	3.87	1.95	0.04087	up
C487	2-[(3S)-1-Benzyl-3-pyrrolidinyl]-1,3-benzothiazole	3.22	294.12	6.21	2.63	0.00884	up
C491	L-Phenylalanine	2.88	165.08	2.06	1.05	0.03819	up
C495	Difenoxurone	4.06	286.14	5.22	2.38	0.02337	up
C503	VMK	3.97	376.21	5.23	2.39	0.00024	up
C517	2-(2-amino-3-methylbutanamido)-3-phenylpropanoic acid	3.36	264.15	4.99	2.32	0.00041	up
C518	Pyroglutamylvaline	1.96	228.11	3.54	1.82	0.01333	up
C535	Desthiobiotin	3.00	214.13	2.57	1.36	0.01598	up
C544	Nalbuphine	4.45	357.19	18.42	4.20	0.03314	up
C554	2-(acetylamino)-3-(1H-indol-3-yl)propanoic acid	3.36	246.10	3.20	1.68	0.04298	up
C558	nylon oligomers amide and group L2	0.58	300.18	9.62	3.27	0.00712	up
C559	Pholcodine	2.96	398.22	5.40	2.43	0.00032	up
C574	Asn-Val-Leu	3.38	344.21	9.45	3.24	0.00120	up
C576	APK	2.82	314.20	12.98	3.70	0.00847	up
C583	Glu-Pro-Leu	3.30	357.19	2.45	1.29	0.04834	up
C608	Val-Ile	2.20	230.16	5.83	2.54	0.02387	up
C623	Alimemazine	5.16	298.15	9.10	3.19	0.02799	up
C629	Promethazine	3.61	284.14	3.05	1.61	0.00971	up
C633	1-Cyclohexyl-3-[(1S,2S,3S,4R,5R)-4-{[2-(dimethylamino)ethyl](methyl)amino}-3-hydroxy-6,8-dioxabicyclo[3.2.1]oct-2-yl]urea	3.77	370.26	10.72	3.42	0.02639	up
C638	Leu-Glu-Thr	3.32	361.19	13.93	3.80	0.00864	up
C644	Ala-Ile-Leu	3.73	315.22	5.09	2.35	0.02238	up
C645	MK-812	3.35	469.25	11.90	3.57	0.00306	up
C658	Ile-Gly-Leu	3.02	301.20	4.05	2.02	0.00743	up
C661	LGLGMAPTXWEBIH-XIXMHUQVSA-N	3.54	268.17	2.89	1.53	0.04364	up
C666	Isoleucyl-Threonine	2.04	232.14	5.01	2.32	0.00737	up
C683	PG(16:0/18:1(11Z))	11.40	748.52	10.36	3.37	0.01769	up
C691	Ala-Leu-Glu	2.85	331.17	6.87	2.78	0.01848	up

C708	Val-Phe-Leu	4.08	377.23	6.25	2.64	0.01237	up
C730	7-Hydroxycoumarinyl arachidonate	7.04	448.26	3.39	1.76	0.03683	up
C735	JWH-018	4.51	341.17	4.45	2.15	0.01829	up
C746	PTI-1	4.89	355.21	8.42	3.07	0.03949	up
C750	1-pentadecanoyl-2-hydroxy-sn-glycero-3-phosphocholine	6.84	481.32	1.73	0.79	0.01012	up
C756	Leu-Gln-Leu	3.24	372.24	8.67	3.12	0.00956	up
C761	2-{2-oxo-2-[(2-oxo-3-azepanyl)amino]ethoxy}acetic acid	0.58	244.11	6.67	2.74	0.00806	up
C762	(E)-3-(4-acetyloxy-2,3-dihydroxy-2,5,5,8a-tetramethyl-3,4,4a,6,7,8-hexahydro-1H-naphthalen-1-yl)prop-2-enoic acid	4.89	354.20	7.21	2.85	0.01996	up
C767	YLK	4.39	422.25	7.71	2.95	0.03526	up
C772	UWNYKRUQYQBDHM-UHFFFAOYSA-N	3.73	270.19	3.89	1.96	0.03376	up
C773	Indolelactic acid	2.83	205.07	2.87	1.52	0.00006	up
C775	Tyr-Ile	3.31	294.16	2.55	1.35	0.02572	up
C781	MPK	3.58	374.20	2.99	1.58	0.04916	up
C786	MG(0:0/22:5(4Z,7Z,10Z,13Z,16Z)/0:0)	7.87	404.29	6.25	2.64	0.01039	up
C808	Pinoxaden	3.36	400.23	7.59	2.92	0.00596	up
C813	Perazine	3.97	339.18	1.86	0.89	0.03441	up
C814	PC O-20:4	6.78	543.33	3.07	1.62	0.00096	up
C819	1,5-Diphenylcarbazide	3.03	242.12	1.84	0.88	0.00397	up
C823	L-Tryptophan	3.30	204.09	1.96	0.97	0.02972	up
C835	Pro-Glu-Arg	3.76	400.21	6.73	2.75	0.01059	up
C842	Pro-Glu	3.36	244.11	8.81	3.14	0.02666	up
C870	WCGQPPIMPLNHFG-YBUISMJYSA-N	4.13	327.18	5.19	2.38	0.03788	up
C896	Irganox 565 degradation product C19H34O1N4S2	3.97	398.22	6.38	2.67	0.01708	up
C903	Lys-Arg-Arg	4.64	458.30	2.87	1.52	0.03860	up
C905	Octaethylene glycol	3.10	370.22	3.75	1.91	0.01761	up
C916	Val-Ile-Gln	3.30	358.22	8.38	3.07	0.00036	up
C927	LysoPC(17:0/0:0)	8.11	509.35	1.71	0.77	0.02655	up
C935	EPK	3.23	372.20	6.61	2.72	0.00239	up
C946	Pro-Thr-Lys	2.21	344.21	6.16	2.62	0.01509	up
C948	3-[(1R,9S)-11-(1-Acetyl-4-piperidinyl)-6-oxo-7,11-diazatricyclo[7.3.1.02,7]trideca-2,4-dien-5- [illegible]	7.59	416.22	3.18	1.67	0.00419	up
C970	1,4-Anhydro-2,5-dideoxy-5-[(isopropylcarbamoyl)amino]-2-(3-oxetanylamino)-D-arabinitol	2.31	273.17	4.14	2.05	0.02393	up
C990	HET0016	1.32	206.14	22.65	4.50	0.02181	up

C998	Leu-Gly-Leu	3.71	301.20	3.64	1.86	0.00801	up
C1052	2-Ethoxy-2-ethyloxanilide	3.90	312.15	3.50	1.81	0.00666	up
C1059	MQENNBZSORVHQZ-XPNMZNARSA-N	4.07	390.23	5.78	2.53	0.00162	up
C1069	Glu-Tyr-Phe	3.85	457.19	8.05	3.01	0.01068	up
C1080	2-[(2R,4aR,8R,8aR)-8-hydroxy-4a,8-dimethyl-decahydronaphthalen-2-yl]prop-2-enoic acid	3.88	252.17	5.22	2.38	0.03329	up
C1096	Leucylphenylalanine	4.02	278.16	2.45	1.29	0.01872	up
C1104	Tributyl trimellitate	4.83	378.20	9.71	3.28	0.01062	up
C1111	Leu-Gly-Gln	1.25	316.17	7.77	2.96	0.00348	up
C1124	A-796260 Degradant	3.26	354.23	5.84	2.55	0.00003	up
C1132	3,3-Dimethyl-1,5-dioxacyclopentadecane-6,15-dione	4.54	270.18	1.52	0.61	0.02957	up
C1148	PC O-20:3	7.04	545.35	2.49	1.32	0.01299	up
C1154	(3R,5S)-1-(1H-Indol-3-ylmethyl)-5-[3-(3-methylphenyl)-1,2,4-oxadiazol-5-yl]-3-pyrrolidinol	4.46	374.17	4.24	2.08	0.04107	up
C1159	Asymmetric dimethylarginine	0.55	202.14	2.32	1.21	0.01960	up
C1166	1-{[(2R,3S,4R,5S)-3,4-Dihydroxy-5-{2-[(2R)-2-(methoxymethyl)-1-pyrrolidinyl]-2-oxoethyl}tetrahydro-2-furanyl]methyl}-3-isopropylurea	3.61	373.22	10.92	3.45	0.02617	up
C1215	[(2R,4S,5S)-5-Ethynyl-1-azabicyclo[2.2.2]oct-2-yl]methyl (2,4-difluorophenyl)carbamate	3.48	320.14	2.19	1.13	0.00460	up
C1229	Capsaicin	2.77	305.20	5.64	2.50	0.02924	up
C1237	5,7-dihydroxy-3-(4-hydroxyphenyl)-4H-chromen-4-one	3.85	270.05	1.56	0.65	0.00899	up
C1250	Pro-Asp-Lys	2.98	358.19	6.95	2.80	0.00061	up
C1256	Ergosterol peroxide	10.41	428.33	3.38	1.76	0.02172	up
C1264	Glu-Arg-Pro	3.65	400.21	28.26	4.82	0.00419	up
C1266	Arg-Ser-Asn	3.81	375.18	3.59	1.85	0.00143	up
C1287	Val-Arg-Pro	4.34	370.24	5.01	2.33	0.01587	up
C1292	Isophorone diamine	4.26	170.18	2.85	1.51	0.02438	up
C1297	Alfuzosin	4.27	389.21	5.12	2.35	0.04459	up
C1334	Testosterone 17-phenylpropionate	7.97	420.26	4.36	2.13	0.03626	up
C1370	Glutamyltryptophan	3.47	333.13	3.58	1.84	0.02475	up
C1388	JWH 073 6-methoxyindole analog	5.32	357.17	38.98	5.28	0.00671	up
C1389	ADBICA	3.24	343.23	3.02	1.59	0.04859	up
C1416	Val-Gly-Ile	3.27	287.18	3.27	1.71	0.02546	up
C1419	octhilinone [ANSI]	3.03	213.12	4.35	2.12	0.00300	up
C1422	2-(1-Adamantyl)-6-{[(5-hydroxy-4-pentyl-4H-1,2,4-triazol-3-yl)thio]methyl}pyrimidin-4-ol	3.08	429.22	5.82	2.54	0.02989	up
C1424	PE(14:0/16:0)	11.00	663.49	7.48	2.90	0.00401	up

C1429	Glycyl-Tryptophan	3.00	261.11	2.16	1.11	0.03729	up
C1451	Tiglylcarnitine	3.27	243.15	22.83	4.51	0.02491	up
C1456	CICCCQGNZFICLV-NTEUORMPSA-N	4.07	258.16	4.08	2.03	0.00483	up
C1476	Ethoxylated trimethylolpropane triacrylate	4.14	428.21	7.99	3.00	0.00019	up
C1479	Dodecyltrimethylammonium	6.37	227.26	2.33	1.22	0.00100	up
C1490	Estrone	4.35	270.16	17.01	4.09	0.00892	up
C1510	3,6,9,12,15,18-Hexaoxaicosane-1,20-diol	3.30	326.20	4.05	2.02	0.00964	up
C1511	Trp-Ile	4.01	317.17	3.90	1.96	0.00643	up
C1514	4-(1-Phenylethyl)-N-[4-(1-phenylethyl)phenyl]benzenamine	4.00	377.22	8.59	3.10	0.00090	up
C1524	UJRMJTIXXKZFGB-KWEBYEANSA-N	7.84	516.38	1.83	0.87	0.00624	up
C1525	5-{2-Hydroxy-3-[(2-methyl-2-propanyl)amino]propoxy}-1,2,3,4-tetrahydronaphthalene-2,3-diyl diacetate	5.31	393.21	6.25	2.64	0.01551	up
C1533	Phe-Glu-Ile	3.86	407.21	2.85	1.51	0.02565	up
C1572	Arg-Arg-Asn	3.72	444.26	9.38	3.23	0.00022	up
C1638	(4-Anilino-1-naphthyl){bis[4-(dimethylamino)phenyl]}methanol	3.50	487.26	13.66	3.77	0.00026	up
C1639	Cholestenone	8.57	384.34	3.26	1.70	0.01988	up
C1640	DUNFAZHEVVYXGG-XTYQBWKXSA-N	3.86	522.17	1.53	0.61	0.00892	up
C1643	Methoxyacetyl fentanyl-d5	3.90	357.25	4.67	2.22	0.01067	up
C1645	2,5,8,11,14,17-Hexaoxanonadecan-19-ol	3.17	296.18	5.73	2.52	0.00731	up
C1650	2,5,8,11,14,17,20,23-Octaoxapentacosan-25-ol	4.18	384.24	11.02	3.46	0.00115	up
C1658	2-{[4-(2,2-Dicyanovinyl)-3-methylphenyl](ethyl)amino}ethyl phenylcarbamate	4.70	374.17	6.33	2.66	0.01675	up
C1664	Cis-4-heptenal	3.12	112.09	1.52	0.61	0.00060	up
C1678	RLK	4.42	415.29	3.79	1.92	0.02450	up
C1701	Dioctylamine	7.10	241.28	1.88	0.91	0.01581	up
C1711	Ile-Val-Leu	4.26	343.25	9.44	3.24	0.00958	up
C1714	Methionyl-Phenylalanine	3.44	296.12	8.78	3.13	0.00014	up
C1715	1,2,2,6,6-Pentamethyl-4-piperidinyl acrylate	4.50	225.17	1.72	0.78	0.02627	up
C1733	gamma-Glutamylphenylalanine	3.45	294.12	7.23	2.85	0.00103	up
C1756	Panthenol	1.63	205.13	2.24	1.16	0.00099	up
C1771	Antioxidant 1135	10.96	390.31	2.85	1.51	0.01523	up
C1794	gamma-Glutamyltyrosine	1.46	310.12	13.79	3.79	0.00039	up
C1803	Val-Gly-Ser	2.43	261.13	2.67	1.42	0.01293	up
C1813	L-alpha-Glutamyl-L-lysine	4.18	275.15	5.83	2.54	0.01256	up
C1814	Val-Gly-Lys	1.48	302.20	13.02	3.70	0.01329	up

C1828	Rolipram	4.05	275.15	6.44	2.69	0.00276	up
C1845	L-Acetylcarnitine	3.20	204.12	4.16	2.06	0.00682	up
C1860	4-Hydroxynonenal	4.44	156.12	1.83	0.87	0.02797	up
C1906	N-(4-Methylphenyl)-N-{2-[4-(methylsulfonyl)-1-piperazinyl]ethyl}ethanediamide	3.10	368.15	1.54	0.63	0.00103	up
C1940	Lys-Ile-Tyr	4.12	422.25	3.61	1.85	0.02554	up
C1958	Arg-Ser-Ala	3.11	332.18	6.08	2.60	0.00511	up
C1973	Isoleucyl-Serine	0.90	218.13	6.49	2.70	0.01869	up
C1976	2-DODECYL-N-(1,2,2,6,6-PENTAMETHYLPIPERIDIN-4-YL)SUCCINIMIDE	9.07	420.37	3.06	1.61	0.01656	up
C1977	Arg-Met-His	3.50	442.21	3.26	1.70	0.04532	up
C1978	4-Aminohippuric acid	2.15	194.07	1.62	0.70	0.00069	up
C1993	β-Hydroxyfentanyl	4.07	352.21	2.47	1.31	0.02792	up
C1997	Drometrizole	4.15	225.09	3.89	1.96	0.00336	up
C2000	Ribothymidine	0.98	258.09	3.06	1.62	0.04423	up
C2064	4-Pyridoxic acid	0.79	183.05	1.81	0.85	0.02396	up
C2081	4-Hydroxybenzaldehyde	3.47	122.04	2.39	1.26	0.01698	up
C2082	2-methoxyhydroquinone	3.47	140.05	2.39	1.26	0.01698	up
C2086	(Hydroxyethyl)methacrylate	2.40	130.06	2.24	1.17	0.01238	up
C2094	(2R)-2,3-Dihydroxypropanoic acid	0.54	106.03	3.70	1.89	0.03906	up
C2119	Tilmicosin	13.03	868.57	2.74	1.45	0.04750	up
C2134	PG O-18:3_16:0	13.03	730.51	7.37	2.88	0.01113	up
C2136	Salicyluric acid	4.02	195.05	1.65	0.73	0.02626	up
C2139	nylon oligomers C4	7.28	450.32	5.26	2.39	0.04302	up
C2149	2-[(2S,3R,4S,5R)-5-(Acetamidomethyl)-3,4-dihydroxytetrahydro-2-furanyl]-N-isopropylacetamide	3.11	274.15	8.83	3.14	0.03047	up
C2199	alpha-Ketoisovaleric acid	1.10	116.05	2.32	1.22	0.01975	up
C2253	PI(16:0/18:2(9Z,12Z))	13.03	834.53	1.71	0.77	0.01428	up
C2276	LPA 20:0	7.52	466.31	3.90	1.96	0.00754	up
C2287	Ile-Asp	1.10	246.12	6.82	2.77	0.00769	up
C2290	PG 4:0_18:0	8.47	582.35	5.33	2.41	0.01832	up
C2314	Leu-Glu-Ile	3.96	373.22	25.15	4.65	0.03740	up
C2315	SAHCQBPGXQFTRA-MTWZWZNHSA-N	6.44	596.30	1.51	0.60	0.00299	up
C2337	gamma-Glutamylvaline	1.39	246.12	6.68	2.74	0.00175	up
C2358	Isoleucyl-Valine	0.59	230.16	11.50	3.52	0.04112	up
C2366	gamma-Glutamylisoleucine	1.36	260.14	6.97	2.80	0.00014	up

C2370	Nicotinuric acid	0.74	180.05	2.14	1.10	0.04113	up
C2379	Valylaspartic acid	0.56	232.11	2.47	1.30	0.04561	up
C2401	Ostruthin	5.16	298.15	8.00	3.00	0.02711	up
C2405	Ile-Phe	3.79	278.16	6.32	2.66	0.02780	up
C2407	UR-144 5-pentanoic acid metabolite	4.58	341.19	17.22	4.11	0.01890	up
C2422	Ala-Ile	2.06	202.13	11.03	3.46	0.02977	up
C2431	Butabarbital	1.78	212.12	8.18	3.03	0.00061	up
C2436	CSRXCVLPMJBQIM-UHFFFAOYSA-N	1.42	242.13	6.70	2.75	0.00145	up
C2437	Ile-Gly	0.94	188.12	8.11	3.02	0.00143	up
C2451	NP-005483	3.23	294.12	10.16	3.34	0.00255	up
C2456	(+)-Dihydrokavain	0.86	232.11	6.78	2.76	0.00062	up
C2472	6-Oxopiperidine-2-carboxylic acid	0.55	143.06	2.10	1.07	0.02076	up
C2482	Glycyl-L-leucine	2.06	188.12	7.51	2.91	0.00145	up
C2494	Pyroglutamylglycine	0.56	186.06	3.09	1.63	0.01417	up
C2513	Ile-Thr	0.90	232.14	9.47	3.24	0.01254	up
C2524	OIFCPZGZZQQDNO-UHFFFAOYSA-N	4.44	200.10	1.71	0.77	0.00893	up
C2536	NP-016437	4.02	284.16	1.80	0.85	0.02546	up
C2541	Ala-Glu-Ile	3.36	331.17	14.68	3.88	0.01301	up
C2545	Ala-Thr-Gln	3.84	318.16	5.96	2.58	0.01600	up
C2567	4-Acetamido-5-[2-(4-hydroxyphenyl)ethylamino]-5-oxopentanoic acid	3.38	308.14	8.36	3.06	0.03846	up
C2568	5-(tert-butyl)-2-methyl-N-(5-methyl-3-isoxazolyl)-3-furamide	3.38	262.13	8.36	3.06	0.03846	up
C2571	11(12)-EET	7.37	320.24	1.81	0.85	0.04101	up
C2579	Glu-Ile-Thr	3.33	361.19	15.27	3.93	0.01047	up
C2591	LPG 18:2	6.62	508.28	2.77	1.47	0.02775	up
C2600	Aminocaproic acid	2.06	131.09	1.87	0.90	0.03802	up
C2616	Cylindrol A1	3.76	430.24	17.00	4.09	0.00781	up
C2623	4-Hydroxyphenylpyruvic acid	4.49	180.04	2.02	1.01	0.00305	up
C2638	VANGVRQDFZGVJU-ZYUHXDNHSA-N	3.86	274.15	10.99	3.46	0.04047	up
C2640	Glu-Val	2.05	246.12	9.76	3.29	0.00029	up
C2653	PG 3:0_16:0	7.54	540.31	4.34	2.12	0.01402	up
C2657	Mephobarbital	3.40	246.10	13.84	3.79	0.02564	up
C2680	Ala-Leu	1.61	202.13	10.83	3.44	0.00069	up
C2683	Gly-Ala-Leu	2.03	259.15	10.12	3.34	0.03709	up

C2695	Val-Phe	3.37	264.15	10.61	3.41	0.00151	up
C2714	5-Methylcytidine	0.76	257.10	3.33	1.73	0.02239	up
C2716	Glu-Glu-Ile	3.39	389.18	4.83	2.27	0.02881	up
C2717	N-Acetylornithine	0.54	174.10	2.18	1.13	0.00427	up
C2722	Met-Glu	1.39	278.09	12.94	3.69	0.00007	up
C2725	Pyridoxine	0.57	169.07	2.23	1.15	0.01197	up
C2729	Ethyl {(1S,4S,5S)-4-[(isonicotinoylamino)methyl]-5-isopropyl-2-methyl-2-cyclohexen-1-yl}acetate	3.69	358.22	11.78	3.56	0.02358	up
C2732	1-({(2R,3S,4R,5S)-3,4-Dihydroxy-5-[2-oxo-2-(1-pyrrolidinyl)ethyl]tetrahydro-2-furanyl}methyl)-3-	3.85	329.20	7.37	2.88	0.00037	up
C2745	Glu-Gln-Ile	3.30	388.20	10.65	3.41	0.00320	up
C2746	Thr-Ile	2.03	232.14	8.03	3.01	0.02871	up
C2750	Val-Ile-Ile	3.85	343.25	7.18	2.84	0.03973	up
C2756	6β-Hydromorphol	1.50	287.15	5.29	2.40	0.00772	up
C2764	Alanylalanine	0.75	160.08	2.61	1.39	0.04960	up
C2766	2-methyl-N-[4-(4-methylpiperazino)benzyl]benzenesulfonamide	3.11	359.17	6.72	2.75	0.00469	up
C2769	Ala-Gln-Thr	4.83	318.16	7.00	2.81	0.03586	up
C2776	KQJGPGHQDDZVHJ-ZZXKWVIFSA-N	0.57	244.13	7.86	2.97	0.00721	up
C2781	Ala-Phe	2.93	236.12	13.20	3.72	0.00580	up
C2784	SKLSOJOIYMIFGM-UHFFFAOYSA-N	1.47	272.10	2.61	1.39	0.02411	up
C2799	LEE	2.86	389.18	8.33	3.06	0.03491	up
C2801	Val-Gly	0.93	174.10	5.70	2.51	0.00092	up
C2805	Cyclo(Leu-Phe)	4.22	260.15	6.91	2.79	0.01683	up
C2806	PG 4:0_17:0	8.12	568.34	5.88	2.56	0.01896	up
C2814	2-[(2-{[(Benzyloxy)carbonyl]amino}-3-methylpentanoyl)amino]acetic acid	3.28	322.15	1.73	0.79	0.03106	up
C2825	Isopropyl 4-hydroxybenzoate	4.38	180.08	2.76	1.47	0.02439	up
C2839	Gln-Leu-Ile	3.71	372.24	15.58	3.96	0.00367	up
C2880	4-(2,7-Dihydroxy-6-methyl-2-heptanyl)-3-hydroxybenzoic acid	4.39	282.15	1.81	0.86	0.04426	up
C2889	Paracetamol	1.47	151.06	2.28	1.19	0.00054	up
C2891	Lys-Val-Pro	3.25	342.23	7.32	2.87	0.00687	up
C2892	3-O-Methylcytidine	1.04	257.10	5.57	2.48	0.01035	up
C2899	Asp-Ser-Arg	3.82	376.16	2.01	1.00	0.01975	up
C2934	2-[(3S)-1-(Cyclohexylmethyl)-3-pyrrolidinyl]-5-fluoro-1H-benzimidazole	2.90	301.20	11.86	3.57	0.00002	up
C2947	Ala-Gln-Leu	2.88	330.19	20.25	4.34	0.00027	up
C2956	Ile-Val-Phe	4.09	377.23	10.03	3.33	0.01654	up

C2957	Phe-Val	3.15	264.15	7.12	2.83	0.01722	up
C2968	Ile-Tyr	3.07	294.16	6.95	2.80	0.00147	up
C2974	N-Formyl-L-methionine	2.24	177.05	2.60	1.38	0.04784	up
C2977	Ile-Gly-Ile	3.44	301.20	11.24	3.49	0.00004	up
C2979	Gln-Val-Ala	0.80	316.17	20.96	4.39	0.00004	up
C2984	N1-(3-Chlorobenzyl)-2-(1-adamantyl)acetamide	2.83	317.16	16.28	4.02	0.00359	up
C2989	[(6Z,10Z)-6-(acetyloxymethyl)-10-(hydroperoxymethyl)-3-methylidene-2-oxo-3a,4,5,8,9,11a-hexahydrocyclodeca[b]furan-4-yl] 2-methylbutanoate	3.62	422.20	14.39	3.85	0.03759	up
C2991	Gln-Glu-Ile	2.88	388.20	11.85	3.57	0.00061	up
C2993	Phe-Gly-Ile	3.60	335.18	10.56	3.40	0.00189	up
C2994	LPG 18:3	6.39	506.26	2.70	1.43	0.00283	up
C3010	Ile-Gln-Pro	3.46	356.21	14.77	3.88	0.01181	up
C3017	Decamethyltetrasiloxane	1.06	310.13	4.01	2.00	0.03604	up
C3023	N-hydroxy-Benzenecarboximidic acid methyl ester	1.26	151.06	1.66	0.73	0.02435	up
C3049	Deoxyadenosine	0.82	251.10	2.46	1.30	0.04531	up
C3068	Vinyl 6,6-dimethylheptanoate	5.10	184.15	2.87	1.52	0.00177	up
C3069	4-[(2S,3S,4S)-4-[4-(β-D-Glucopyranosyloxy)-3-methoxybenzyl]-3-(hydroxymethyl)tetrahydro-2-furanyl]-2-methoxyphenyl β-D-glucopyranoside	6.08	684.27	2.53	1.34	0.00242	up
C3075	L-Aspartic acid	0.54	133.04	3.14	1.65	0.04076	up
C3080	Thr-Ala-Ile	2.94	303.18	7.10	2.83	0.01919	up
C3092	1,3-Dicyclohexylurea	4.51	224.19	10.26	3.36	0.02680	up
C3099	Glu-Ile-Ile	4.07	373.22	12.71	3.67	0.00057	up
C3100	Asn-Glu-Leu	2.91	374.18	19.76	4.30	0.00058	up
C3117	LPI 18:3	6.13	594.28	2.09	1.06	0.00119	up
C3128	Paracetamol sulfate	2.78	231.02	1.71	0.78	0.02588	up
C3143	1-AMINOCYCLOPROPANE-1-CARBOXYLATE	0.79	101.05	3.29	1.72	0.03083	up
C3145	LPG O-24:6	7.77	570.34	6.89	2.78	0.04693	up
C3149	Leu-Leu-Ile	4.09	357.26	9.98	3.32	0.03351	up
C3159	Bisphenol AP	3.65	290.13	9.65	3.27	0.03360	up
C3170	N-{4-[(R)-[(3R)-4-(4-Fluorobenzyl)-5-oxo-3-morpholinyl](hydroxy)methyl]phenyl}acetamide	4.57	372.16	6.27	2.65	0.01879	up
C3171	Thr-Thr-Leu	2.97	333.19	31.75	4.99	0.02739	up
C3175	Tiapride	3.53	328.14	11.97	3.58	0.00948	up
C3185	Gln-Gly-Leu	2.64	316.17	22.05	4.46	0.00109	up

C3188	Thr-Val-Ile	3.48	331.21	14.13	3.82	0.00049	up
C3193	Oxydi-1,2-propanediyl tetraphenyl bis(phosphite)	3.35	566.16	1.55	0.64	0.03937	up
C3195	Val-Thr-Leu	2.89	331.21	18.27	4.19	0.00063	up
C3202	LNAPE 3:0/N-19:1	7.98	549.34	2.13	1.09	0.03689	up
C3205	NP-024154	2.87	302.11	8.30	3.05	0.00031	up
C3213	Ser-Arg-Asn	4.47	375.18	10.24	3.36	0.00409	up
C3218	Amorfrutin 4	5.31	394.22	4.04	2.01	0.03075	up
C3219	Citral	3.69	152.12	1.76	0.81	0.03693	up
C3250	CIGSWLXZMSXAAE-ZCFIWIBFSA-N	4.18	274.10	7.47	2.90	0.02453	up
C3251	Thallium(3+) tris(6-methyl-1-heptanolate)	7.16	592.36	2.44	1.29	0.03795	up
C3263	Pro-Ala-Thr	3.30	287.15	13.93	3.80	0.00327	up
C3298	Hexaethyldisiloxane	4.71	246.18	2.29	1.19	0.01104	up
C3304	Gln-Gln-Leu	2.95	387.21	27.08	4.76	0.01384	up
C8	Choline	0.52	103.10	0.51	-0.96	0.01764	down
C44	Palmitoyl ethanolamide	8.29	299.28	0.34	-1.56	0.02862	down
C45	Emetine	6.74	480.30	0.38	-1.41	0.00326	down
C101	Stearoyl Ethanolamide	9.07	327.31	0.21	-2.23	0.02786	down
C104	Dicyclomine	6.87	309.27	0.12	-3.01	0.00388	down
C107	Stigmasterol	12.98	412.37	0.59	-0.76	0.00583	down
C134	Hypoxanthine	0.56	136.04	0.08	-3.59	0.01769	down
C168	Aliskiren	8.92	551.40	0.45	-1.14	0.00469	down
C180	PI-Cer 34:0;3O	13.08	797.54	0.14	-2.79	0.04979	down
C183	Pyrogallol	2.14	126.03	0.10	-3.28	0.03297	down
C197	1,1-Bi(cyclohexyl)	8.10	166.17	0.23	-2.10	0.00327	down
C253	NAGly 15:0/16:0	12.88	553.47	0.41	-1.30	0.04436	down
C290	7-(Diethylamino)-4-methyl-2H-chromen-2-one	6.63	231.13	0.59	-0.77	0.00119	down
C295	Stearoylcarnitine	8.49	427.37	0.38	-1.40	0.02232	down
C313	PI-Cer 35:0;3O	13.08	811.56	0.11	-3.17	0.00375	down
C320	LysoPC(26:0/0:0)	13.05	635.49	0.10	-3.35	0.03547	down
C329	Nicotinic acid ribonucleoside	0.52	256.08	0.46	-1.13	0.03013	down
C376	N-(2-Hydroxyethyl)-9-octadecenamide	8.80	325.30	0.12	-3.01	0.03666	down
C445	N,N-Dimethyloctadecanamide	9.96	311.32	0.64	-0.64	0.04281	down
C472	QOLRLLFJMZLYQJ-KVERTZGMSA-N	7.19	430.30	0.18	-2.47	0.00063	down

C511	Ethoxyquin	6.03	217.15	0.64	-0.65	0.01734	down
C521	PI-Cer 33:0;3O	13.09	783.53	0.08	-3.63	0.01567	down
C525	PC(18:0/P-18:1(11Z))	9.23	771.62	0.45	-1.16	0.01823	down
C536	Doxepin	3.85	279.16	0.37	-1.42	0.02670	down
C538	LysoPI(18:0/0:0)	7.41	600.33	0.51	-0.98	0.03839	down
C564	NP-008952	4.94	228.14	0.32	-1.66	0.00457	down
C580	Cer 20:0;2O/2:0	8.34	371.34	0.37	-1.43	0.00053	down
C595	HexCer 18:1;2O/16:0	11.95	699.56	0.29	-1.79	0.01893	down
C649	Cholest-4-en-3-one	11.88	384.34	0.45	-1.16	0.02218	down
C706	Methyl caffeate	3.76	194.06	0.49	-1.04	0.00319	down
C780	FUB-144	0.48	349.18	0.55	-0.87	0.01081	down
C867	Cer 18:0;2O/18:2	12.73	563.53	0.50	-1.01	0.04033	down
C902	Pentadecanoyl Ethanolamide	7.86	285.27	0.13	-2.95	0.00694	down
C911	Myristoyl Ethanolamide	7.43	271.25	0.61	-0.72	0.03602	down
C920	N-Isobutyl-2,4,12-octadecatrienamide	9.60	333.30	0.52	-0.95	0.02708	down
C968	Diisodecyl phthalate	8.98	446.34	0.09	-3.43	0.03109	down
C972	2-Arachidonoyl glycerol	9.26	378.28	0.52	-0.95	0.00383	down
C979	Erucic acid	10.51	338.32	0.46	-1.13	0.00429	down
C1013	Mesitaldehyde	3.78	148.09	0.63	-0.68	0.01483	down
C1017	Cer 17:0;2O/2:0	7.17	329.29	0.10	-3.38	0.03636	down
C1061	MG(0:0/14:1(9Z)/0:0)	6.25	300.23	0.34	-1.56	0.01575	down
C1079	Phenylalanylthreonine	3.55	266.13	0.37	-1.43	0.02350	down
C1086	Pro-Arg-Ala	3.90	342.20	0.17	-2.53	0.00160	down
C1110	N,N-Diphenylguanidine	3.74	211.11	0.41	-1.29	0.00482	down
C1129	METHYL OLEATE	9.54	296.27	0.60	-0.73	0.02068	down
C1135	VCJZLHSIECUOIT-NSHDSACASA-N	4.62	232.12	0.27	-1.87	0.01080	down
C1200	4,8-Dihydroxy-7-(hydroxymethyl)-6-methoxy-3,4-dihydro-1(2H)-naphthalenone	4.65	238.08	0.13	-3.00	0.00024	down
C1201	Irganox 1010 degradation product C22H36O6	6.07	396.25	0.56	-0.84	0.00947	down
C1203	Tyr-Ala	3.22	252.11	0.28	-1.83	0.02140	down
C1209	Dibutyl sebacate	8.01	314.25	0.65	-0.62	0.00571	down
C1213	2-{5-[2-({2-[5-(2-Hydroxypropyl)tetrahydro-2-furanyl]propanoyl}oxy)propyl]tetrahydro-2-furanyl}propanoic acid	4.33	386.23	0.35	-1.51	0.00409	down
C1221	4-Acetylphenetole	8.93	164.08	0.40	-1.33	0.00335	down

C1235	4-fluoro-3-methyl-alpha-Pyrrolidinovalerophenone	3.04	263.17	0.47	-1.09	0.03075	down
C1252	NP-015980	4.08	288.16	0.26	-1.96	0.03418	down
C1281	diethyl 3-amino-6-methylthieno[2,3-b]pyridine-2,5-dicarboxylate	4.63	308.08	0.52	-0.95	0.00797	down
C1293	NP-017176	9.33	444.28	0.58	-0.79	0.01702	down
C1301	Tris(2-ethylhexyl) phosphite	8.38	418.36	0.64	-0.65	0.02635	down
C1356	Methyl N-{[(2S)-1-(cyclohexylcarbamoyl)-4-(4-piperidinylcarbonyl)-2-piperazinyl]carbonyl}-D-alaninate	7.40	451.28	0.50	-1.00	0.00047	down
C1375	2,2-ETHYLIDENEBIS(4,6-DI-TERT-BUTYLPHENOL)	4.01	438.35	0.34	-1.56	0.01381	down
C1382	Ethylbenzoate	9.24	150.07	0.11	-3.21	0.01591	down
C1404	Tris(2-butoxyethyl) phosphate	7.57	398.24	0.16	-2.64	0.00354	down
C1423	NP-021844	3.68	248.16	0.09	-3.48	0.03331	down
C1450	8-Isoprostaglandin F1β	7.03	356.26	0.66	-0.60	0.03522	down
C1504	6-Amino-5,6,7,8-tetrahydronaphthalene-2,3-diyl diacetate	3.87	263.12	0.41	-1.29	0.04792	down
C1535	Dihexyl azelaate	9.82	356.29	0.57	-0.80	0.01026	down
C1560	KM 233	4.33	362.23	0.52	-0.95	0.03924	down
C1566	Allyl 3-phenylpropanoate	4.38	190.10	0.52	-0.95	0.02452	down
C1576	Cer 18:1;2O/20:3	12.31	587.53	0.18	-2.44	0.01251	down
C1592	Cys-Thr-His	4.83	416.15	0.57	-0.81	0.04913	down
C1613	3,9-Bis[(11-methyldodecyl)oxy]-2,4,8,10-tetraoxa-3,9-diphosphaspiro[5.5]undecane	8.20	592.40	0.11	-3.23	0.00218	down
C1618	N-(2-Furylmethyl)-3-{[5-(2-methyl-2-propanyl)-1,2-oxazol-3-yl]methyl}-3-oxetanamine	3.90	290.16	0.41	-1.30	0.02419	down
C1627	2-(1H-indol-3-yl)acetic acid	2.89	175.06	0.46	-1.14	0.04974	down
C1651	Ethyl-2-hydroxyethyl nonanedioic acid	3.86	276.16	0.39	-1.36	0.00165	down
C1686	Lys-Gly-Arg	3.56	359.23	0.20	-2.31	0.01124	down
C1697	triisononyl trimellitate	10.08	588.44	0.43	-1.20	0.02921	down
C1716	Sulfolithocholic acid	10.66	456.25	0.22	-2.18	0.01621	down
C1747	5-[5-(methoxycarbonyl)-5,8a-dimethyl-2-methylidene-decahydronaphthalen-1-yl]-3-methylpentanoic acid	5.41	350.25	0.41	-1.30	0.00276	down
C1766	Cer 8:0;3O/26:0;(2OH)	11.70	571.52	0.31	-1.69	0.04010	down
C1767	NLK	3.43	373.23	0.46	-1.12	0.02969	down
C1839	Metamitron	4.00	202.08	0.50	-1.01	0.00434	down
C1865	Aniline Yellow	3.71	197.10	0.37	-1.43	0.02753	down
C1900	7α-Hydroxytestosterone	4.94	304.20	0.32	-1.64	0.02657	down
C1912	Penbutolol	4.90	291.22	0.56	-0.84	0.04764	down
C1941	Arg-Asn	3.89	288.16	0.44	-1.17	0.02547	down
C1950	4-Octyl-N-(4-octylphenyl)aniline	10.48	393.34	0.65	-0.61	0.03359	down

C1952	tween X12	3.89	874.55	0.31	-1.68	0.01434	down
C1975	Glu-Val-Asn	3.58	360.17	0.33	-1.59	0.03630	down
C2011	Diisononyl adipate	9.85	398.34	0.28	-1.86	0.01627	down
C2017	Succinic acid	0.83	118.03	0.08	-3.58	0.02099	down
C2022	ST 29:1;O;S	9.47	494.34	0.43	-1.23	0.00934	down
C2023	ST 28:1;O;S	9.35	480.33	0.54	-0.89	0.01609	down
C2061	PI-Cer 12:0;2O/22:0;O	13.08	797.54	0.21	-2.23	0.02226	down
C2076	Azelaic acid	4.21	188.10	0.61	-0.72	0.03396	down
C2077	11(E)-Eicosenoic Acid	9.82	310.29	0.60	-0.74	0.02626	down
C2078	2-Butoxyethyl (9E)-9-octadecenoate	10.57	382.34	0.38	-1.38	0.00304	down
C2091	PI-Cer 12:0;2O/23:0;O	13.07	811.56	0.17	-2.60	0.00037	down
C2099	ST 29:2;O;S	9.16	492.33	0.37	-1.45	0.00586	down
C2121	1,3-Dioxolane	0.84	74.04	0.11	-3.20	0.01627	down
C2128	Docosanoic Acid	11.56	340.33	0.62	-0.68	0.02341	down
C2163	D&C Red Dye 27	11.37	779.56	0.21	-2.22	0.00939	down
C2170	PI-Cer 12:0;2O/21:0;O	13.08	783.53	0.19	-2.39	0.00386	down
C2223	BTB13021	0.51	220.11	0.47	-1.10	0.02515	down
C2245	NAGlySer 15:0/17:0	12.51	654.52	0.23	-2.10	0.00724	down
C2249	NP-018716	4.98	216.14	0.30	-1.72	0.00807	down
C2261	Nervonic acid	11.45	366.35	0.57	-0.80	0.04519	down
C2266	NAGlySer 15:0/16:0	12.01	640.50	0.20	-2.29	0.00364	down
C2319	13Z,16Z-Docosadienoic Acid	9.88	336.30	0.47	-1.08	0.01607	down
C2336	PI-Cer 12:0;2O/24:0;O	13.06	825.57	0.08	-3.70	0.00028	down
C2454	SL 18:1;O/15:0	9.66	587.46	0.52	-0.96	0.01739	down
C2468	5-Methoxy-2-{[(2S)-2-{3-[2-(2-methoxyethoxy)-3-pyridinyl]-1,2,4-oxadiazol-5-yl}-1- [illegible]	4.23	426.19	0.37	-1.44	0.02415	down
C2509	LNAPE 15:0/N-2:0	6.66	481.28	0.37	-1.42	0.00916	down
C2519	MG(0:0/20:0/0:0)	10.17	386.34	0.40	-1.34	0.00944	down
C2553	Hexadecanedioic acid	5.42	286.21	0.42	-1.23	0.01121	down
C2558	PC(14:0/18:3(6Z,9Z,12Z))	12.01	727.52	0.23	-2.12	0.03235	down
C2590	KQGVEZGOGSMUMX-KTKRTIGZSA-N	9.82	374.32	0.49	-1.03	0.04533	down
C2639	3-Coumaric acid	3.73	164.05	0.64	-0.65	0.00429	down
C2661	NAGlySer 14:0/16:0	11.58	626.49	0.18	-2.50	0.01381	down
C2701	Gly-Asp-Gln	4.01	318.11	0.65	-0.62	0.00399	down

C2713	4-(4-methoxyphenyl)-2-(methylanilino)-6-oxo-1,6-dihydropyrimidine-5-carbonitrile	3.59	332.13	0.50	-0.99	0.04789	down
C2726	NP-017624	4.31	436.21	0.47	-1.08	0.02003	down
C2779	Acoric acid	5.47	268.17	0.28	-1.85	0.00022	down
C2810	LPI 15:0	6.26	558.28	0.20	-2.29	0.03506	down
C2840	(+/-)-C75	5.39	254.15	0.36	-1.46	0.00800	down
C2877	Myristic acid alkyne	6.25	224.18	0.09	-3.44	0.01839	down
C2895	PE O-16:4_15:0	12.42	655.45	0.14	-2.89	0.00014	down
C2954	1-Methyladenine	0.66	149.07	0.35	-1.53	0.00519	down
C2981	SHexCer 42:0;3O	11.34	909.66	0.66	-0.59	0.04274	down
C3037	2,4,8,10-Tetraoxaspiro[5.5]undecane-3,9-diylbis-2-methyl-2,1-propanediyl bis{3-[4-hydroxy-3-methyl-5-(2-methyl-2-propanyl)phenyl]propanoate}	9.50	740.45	0.14	-2.82	0.00021	down
C3047	GFYNKSLVMQVEBT-BAUVLOOGSA-N	7.23	338.25	0.36	-1.49	0.04758	down
C3082	Cer 17:0;2O/16:0;(3OH)	11.71	541.51	0.11	-3.16	0.00651	down
C3144	3-[(4,6-dimethylpyrimidin-2-yl)amino]-2-[(2-methyl-3-furyl)carbonyl]acrylonitrile	3.35	282.11	0.41	-1.29	0.00702	down
C3204	Cer 17:0;2O/15:0;(3OH)	11.30	527.49	0.22	-2.21	0.00562	down
C3277	Irganox 1010 degradation product C41H60O10	8.96	712.42	0.09	-3.52	0.00002	down
C3279	N-Phenylacetylglutamic acid	3.86	265.10	0.43	-1.23	0.02232	down

Supplementary Table S2. Differential metabolites information of TIAP group compared with model group.

ID	Description	Retention_time (min)	m/z	FC	log2FC	Pvalue	Up.Down
C66	Tryptophyl-Valine	3.44	303.16	2.56	1.36	0.00730	up
C170	(4aS,9aR)-2-[(4-Methoxyphenyl)acetyl]-7-[2-(4-morpholinyl)ethyl]decahydro-6H-pyrido[3,4-d]azepin-6-	3.23	429.26	2.60	1.38	0.03596	up
C335	Tri-o-Cresyl phosphate	7.87	368.12	2.87	1.52	0.00764	up
C456	Indoline	3.21	119.07	2.38	1.25	0.04277	up
C487	2-[(3S)-1-Benzyl-3-pyrrolidinyl]-1,3-benzothiazole	3.22	294.12	2.28	1.19	0.02275	up
C545	Metolachlor morpholinone	4.29	233.14	3.07	1.62	0.00935	up
C644	Ala-Ile-Leu	3.73	315.22	2.29	1.20	0.03256	up
C948	3-[(1R,9S)-11-(1-Acetyl-4-piperidinyl)-6-oxo-7,11-diazatricyclo[7.3.1.02,7]trideca-2,4-dien-5- [illegible]	7.59	416.22	3.60	1.85	0.00167	up
C1075	4-Methoxyacetophenone	7.72	150.07	4.34	2.12	0.00052	up
C1132	3,3-Dimethyl-1,5-dioxacyclopentadecane-6,15-dione	4.54	270.18	1.60	0.68	0.03828	up
C1217	Indoleacrylic acid	2.09	187.06	1.97	0.98	0.04242	up
C1292	Isophorone diamine	4.26	170.18	2.39	1.26	0.02014	up
C1437	Gly-Val-Phe	3.77	321.17	1.92	0.94	0.01475	up
C1464	Arachidonic acid	8.64	304.24	1.94	0.95	0.04376	up
C1479	Dodecyltrimethylammonium	6.37	227.26	2.57	1.36	0.01744	up
C1524	UJRMJTIXXKZFGB-KWEBYEANSA-N	7.84	516.38	2.87	1.52	0.04407	up
C1572	Arg-Arg-Asn	3.72	444.26	2.60	1.38	0.02635	up
C1701	Dioctylamine	7.10	241.28	1.93	0.95	0.01254	up
C1715	1,2,2,6,6-Pentamethyl-4-piperidinyl acrylate	4.50	225.17	2.42	1.28	0.04797	up
C1733	gamma-Glutamylphenylalanine	3.45	294.12	2.43	1.28	0.03595	up
C2165	Diethylene glycol monoacetate	0.57	148.07	3.49	1.80	0.02013	up
C2253	PI(16:0/18:2(9Z,12Z))	13.03	834.53	1.83	0.87	0.02882	up
C2364	Histidylglycine	0.56	212.09	2.43	1.28	0.03114	up
C2422	Ala-Ile	2.06	202.13	1.70	0.77	0.02548	up
C2431	Butabarbital	1.78	212.12	1.71	0.78	0.02616	up
C2482	Glycyl-L-leucine	2.06	188.12	2.41	1.27	0.02879	up
C2576	Methyl (1S,4aR,6S,7R,7aS)-1-(-D-glucopyranosyloxy)-4a,7-dihydroxy-6-{[(2E)-3-(4-hydroxyphenyl)-2-propenoyl]oxy}-7-methyl-1,4a,5,6,7,7a-hexahydrocyclopenta[c]pyran-4-carboxylate	3.98	568.18	6.90	2.79	0.01039	up
C2657	Mephobarbital	3.40	246.10	3.36	1.75	0.02050	up

C2684	CMPF	3.66	240.10	1.76	0.82	0.03245	up
C2774	3,14-Dihydroxycarda-5,20(22)-dienolide	8.64	372.23	1.82	0.86	0.00664	up
C2871	AMIDOSULFOBETAINE [ASB-14]	9.41	434.32	1.68	0.75	0.04244	up
C3069	4-[(2S,3S,4S)-4-[4-D-Glucopyranosyloxy)-3-methoxybenzyl]-3-(hydroxymethyl)tetrahydro-2-furanyl]-2-methoxyphenyl D-glucopyranoside	6.08	684.27	1.63	0.71	0.04083	up
C3168	NP-018606	6.31	550.30	2.90	1.54	0.02419	up
C3235	N-[(1R,2S,3R,5R)-2,3-Dihydroxy-5-(3-pyridinyl)cyclopentyl]-2-methylbenzamide	0.59	312.14	3.00	1.58	0.00514	up
C3245	Pentacosanoic acid	9.12	382.38	3.46	1.79	0.02024	up
C41	1,4-dihydroxyheptadec-16-en-2-yl acetate	6.94	328.26	0.27	-1.87	0.00092	down
C107	Stigmasterol	12.98	412.37	0.65	-0.63	0.03155	down
C148	Pinolenic acid	6.94	278.22	0.31	-1.68	0.00166	down
C160	6-[(dimethylamino)methylidene]-6,7,8,9-tetrahydro-5H-benzo[a]cyclohepten-5-one	1.13	215.13	0.50	-1.01	0.02425	down
C230	9-Oxo-ODE	7.38	294.22	0.60	-0.73	0.00598	down
C262	13-HODE	6.34	296.24	0.25	-2.01	0.02295	down
C304	(2S,5aS,8aR)-1-Methyl-6-(4-methylbenzyl)-2-[3-(4-morpholinyl)-3-oxopropyl]octahydropyrrolo[3,2-E][1,4]diazepin-5(2H)-one	4.62	414.26	0.12	-3.05	0.03091	down
C357	SPK	1.36	330.19	0.35	-1.52	0.00069	down
C422	4-hydroxy-6-[2-(2-methyl-1,2,4a,5,6,7,8,8a-octahydronaphthalen-1-yl)ethyl]oxan-2-one	9.33	292.20	0.62	-0.69	0.00351	down
C564	NP-008952	4.94	228.14	0.20	-2.32	0.00344	down
C599	Linoleic acid	9.15	280.24	0.60	-0.73	0.03794	down
C673	trans-2-Dodecenoylcarnitine	7.88	341.26	0.61	-0.71	0.02012	down
C697	Tetradecanoylcarnitine	6.60	371.30	0.34	-1.57	0.01804	down
C706	Methyl caffeate	3.76	194.06	0.58	-0.78	0.02651	down
C743	2-(3-hydroxy-3,7,11,15-tetramethylhexadecyl)-3,5,6-trimethylcyclohexa-2,5-diene-1,4-dione	11.84	446.38	0.59	-0.76	0.03028	down
C816	Riboflavin	3.38	376.14	0.58	-0.79	0.00945	down
C890	Sedanolide	3.63	194.13	0.35	-1.50	0.00380	down
C902	Pentadecanoyl Ethanolamide	7.86	285.27	0.27	-1.88	0.01364	down
C972	2-Arachidonoyl glycerol	9.26	378.28	0.50	-0.99	0.03044	down
C978	(2E,4E,14E)-13-Hydroperoxy-N-(2-methylpropyl)icosa-2,4,14-trienamide	8.69	393.32	0.40	-1.30	0.00240	down
C999	(Vinyloxy)cyclohexane	4.21	126.10	0.42	-1.24	0.02435	down
C1061	MG(0:0/14:1(9Z)/0:0)	6.25	300.23	0.11	-3.24	0.00075	down
C1084	NP-011223	6.92	186.13	0.27	-1.91	0.00492	down
C1086	Pro-Arg-Ala	3.90	342.20	0.36	-1.48	0.02116	down

C1127	2,2-Methylenebis[4-methyl-6-(1-methylcyclohexyl)phenol]	8.08	420.30	0.49	-1.03	0.01615	down
C1149	2,5-Bis(tert-butylperoxy)-2,5-dimethylhexane	7.26	290.25	0.26	-1.92	0.01088	down
C1156	Avocadyne 1-acetate	6.71	326.25	0.32	-1.63	0.00670	down
C1221	4-Acetylphenetole	8.93	164.08	0.56	-0.84	0.03057	down
C1301	Tris(2-ethylhexyl) phosphite	8.38	418.36	0.49	-1.02	0.01250	down
C1384	11-(2,5-Dioxo-2,5-dihydro-1H-pyrrol-1-yl)undecanoic acid	4.23	281.16	0.40	-1.34	0.01847	down
C1413	1,4-Bis(1-propen-1-yloxy)cyclohexane	6.94	196.15	0.41	-1.30	0.03917	down
C1423	NP-021844	3.68	248.16	0.06	-4.00	0.03043	down
C1450	8-Isoprostaglandin F1	7.03	356.26	0.31	-1.68	0.00042	down
C1480	2,6-Dimethyl-4-nonylphenol	8.27	248.21	0.35	-1.52	0.00903	down
C1613	3,9-Bis[(11-methyldodecyl)oxy]-2,4,8,10-tetraoxa-3,9-diphosphaspiro[5.5]undecane	8.20	592.40	0.36	-1.46	0.03226	down
C1651	Ethyl-2-hydroxyethyl nonanedioic acid	3.86	276.16	0.21	-2.24	0.00114	down
C1686	Lys-Gly-Arg	3.56	359.23	0.35	-1.50	0.02994	down
C1691	Ethyl 4-(dimethylamino)butanoate	3.16	159.13	0.62	-0.68	0.02125	down
C1747	5-[5-(methoxycarbonyl)-5,8a-dimethyl-2-methylidene-decahydronaphthalen-1-yl]-3-methylpentanoic acid	5.41	350.25	0.48	-1.07	0.00824	down
C1767	NLK	3.43	373.23	0.55	-0.85	0.04136	down
C1799	DG(16:0/0:0/18:3n6)	11.24	590.49	0.38	-1.40	0.01638	down
C1865	Aniline Yellow	3.71	197.10	0.51	-0.97	0.04027	down
C1869	1,2-Dihydroxyheptadec-16-yn-4-one	7.93	282.22	0.36	-1.49	0.03798	down
C1877	SVEGOFXIARNYNN-VOTSOKGWSA-N	4.42	298.18	0.55	-0.86	0.04987	down
C1898	1-O-benzyl 6-O-(2-ethylhexyl) hexanedioate	7.25	348.23	0.42	-1.26	0.03886	down
C1905	Methionyl-Threonine	6.97	250.10	0.64	-0.64	0.03387	down
C1912	Penbutolol	4.90	291.22	0.38	-1.41	0.01438	down
C2076	Azelaic acid	4.21	188.10	0.45	-1.14	0.01105	down
C2249	NP-018716	4.98	216.14	0.20	-2.31	0.00335	down
C2468	5-Methoxy-2-{[(2S)-2-{3-[2-(2-methoxyethoxy)-3-pyridinyl]-1,2,4-oxadiazol-5-yl}-1- [illegible]	4.23	426.19	0.26	-1.94	0.00956	down
C2553	Hexadecanedioic acid	5.42	286.21	0.40	-1.31	0.01198	down
C2693	13,14-dihydro-15-keto-tetranor Prostaglandin F1?	5.34	300.19	0.29	-1.81	0.00310	down
C2780	RJIZCWQDPRRTRM-UHFFFAOYSA-N	4.22	144.12	0.40	-1.31	0.02458	down
C2840	(+/-)-C75	5.39	254.15	0.49	-1.02	0.00810	down
C2978	15-Crown-5,1,4,7,10,13-Pentaoxa-cyclo-pentadecane	3.86	220.13	0.38	-1.41	0.01367	down
C3037	2,4,8,10-Tetraoxaspiro[5.5]undecane-3,9-diylbis-2-methyl-2,1-propanediyl bis{3-[4-hydroxy-3-methyl-5-(2-methyl-2-propanyl)phenyl]propanoate}	9.50	740.45	0.21	-2.22	0.00059	down

C3048	Corticosterone	6.43	346.22	0.20	-2.35	0.02573	down
C3056	LPI 20:3	6.68	622.31	0.32	-1.64	0.00642	down
C3141	2-[(2S,3R,4S,5R)-3,4-Dihydroxy-5-{[(isopropylcarbamoyl)amino]methyl}tetrahydro-2-furanyl]-N-[2-(dimethylamino)ethyl]acetamide	6.32	346.22	0.21	-2.26	0.03905	down
C3144	3-[(4,6-dimethylpyrimidin-2-yl)amino]-2-[(2-methyl-3-furyl)carbonyl]acrylonitrile	3.35	282.11	0.27	-1.89	0.01132	down
C3163	D-Mannose 1-phosphate	0.50	260.03	0.55	-0.88	0.02184	down
C3267	4-[(Morpholin-4-ylcarbothioyl)sulfanyl]morpholine	0.53	248.07	0.55	-0.88	0.02184	down
C3269	Ethyl benzoylformate	5.23	178.06	0.43	-1.22	0.03271	down
C3277	Irganox 1010 degradation product C41H60O10	8.96	712.42	0.14	-2.79	0.00010	down
